# Effect of low-to-moderate hyperoxia on lung injury in preclinical animal models: a systematic review and meta-analysis

**DOI:** 10.1186/s40635-023-00501-x

**Published:** 2023-04-24

**Authors:** Samuel Minkove, Rhea Dhamapurkar, Xizhong Cui, Yan Li, Junfeng Sun, Diane Cooper, Peter Q. Eichacker, Parizad Torabi-Parizi

**Affiliations:** 1 Critical Care Medicine Department, National Institutes of Health, Building 10, Room 2C145, 9000 Rockville Pike, Bethesda, MD 20892 USA; 2 NIH Library, Clinical Center, National Institutes of Health, Bethesda, MD 20892 USA

**Keywords:** Oxygen toxicity, Oxygen therapy, Animal models, Inflammatory lung injury

## Abstract

**Background:**

Extensive animal investigation informed clinical practice regarding the harmful effects of high fractional inspired oxygen concentrations (FiO_2_s > 0.60). Since questions persist whether lower but still supraphysiologic FiO_2_ ≤ 0.60 and > 0.21 (FiO_2_ ≤ 0.60/ > 0.21) are also harmful with inflammatory lung injury in patients, we performed a systematic review examining this question in animal models.

**Methods:**

Studies retrieved from systematic literature searches of three databases, that compared the effects of exposure to FiO_2_ ≤ 0.60/ > 0.21 vs. FiO_2_ = 0.21 for ≥ 24 h in adult in vivo animal models including an inflammatory challenge or not were analyzed. Survival, body weight and/or lung injury measures were included in meta-analysis if reported in ≥ 3 studies.

**Results:**

More than 600 retrieved reports investigated only FiO_2_s > 0.60 and were not analyzed. Ten studies with an inflammatory challenge (6 infectious and 4 noninfectious) and 14 studies without, investigated FiO_2_s ≤ 0.60/ > 0.21 and were analyzed separately. In seven studies with an inflammatory challenge, compared to FiO_2_ = 0.21, FiO_2_ ≤ 0.60/ > 0.21 had consistent effects across animal types on the overall odds ratio of survival (95%CI) that was on the side of harm but not significant [0.68 (0.38,1.23), *p* = 0.21; *I*^2^ = 0%, *p* = 0.57]. However, oxygen exposure times were only 1d in 4 studies and 2–4d in another. In a trend approaching significance, FiO_2_ ≤ 0.60/ > 0.21 with an inflammatory challenge consistently increased the standardized mean difference (95%CI) (SMD) in lung weights [0.47 (− 0.07,1.00), *p* = 0.09; *I*^2^ = 0%, *p* = 0.50; *n* = 4 studies] but had inconsistent effects on lung lavage protein concentrations (*n* = 3), lung pathology scores (*n* = 4) and/or arterial oxygenation (*n* = 4) (*I*^2^ ≥ 43%, *p* ≤ 0.17). Studies without an inflammatory challenge had consistent effects on lung lavage protein concentration (*n* = 3) SMDs on the side of being increased that was not significant [0.43 (− 0.23,1.09), *p* = 0.20; *I*^2^ = 0%, *p* = 0.40] but had inconsistent effects on body and lung weights (*n* = 6 and 8 studies, respectively) (*I*^2^ ≥ 71%, *p* < 0.01). Quality of evidence for studies was weak.

**Interpretation:**

Limited animal studies have investigated FiO_2_ ≤ 0.60/ > 0.21 with clinically relevant models and endpoints but suggest even these lower FiO_2_s may be injurious. Given the influence animal studies examining FiO_2_ > 0.60 have had on clinical practice, additional ones investigating FiO_2_ ≤ 0.60/ > 0.21 appear warranted, particularly in pneumonia models.

**Supplementary Information:**

The online version contains supplementary material available at 10.1186/s40635-023-00501-x.

## Background

In the early 1900s, Karsner et al. demonstrated that rabbits breathing fractional inspired oxygen concentrations (FiO_2_) of 0.80 for 7 day developed fibrinous bronchopneumonia [1]. Numerous pre-clinical studies subsequently confirmed this relationship between exposure to similar high oxygen concentrations and lung injury [1–8]. This substantial body of preclinical investigation informed recommendations that high FiO_2_s be avoided whenever possible [9–13]. Although recent guidelines and study protocols frequently describe the minimal arterial oxygen saturation (SaO_2_) or pressure (PaO_2_) levels that should be maintained in patients, when stipulated, FiO_2_ targets are typically set to not exceed 0.50 to 0.60 when possible [9–16].

Concern has grown that even low oxygen levels that are still greater than room air (that is FiO_2_s ≤ 0.60 but > 0.21, termed supraphysiologic here) may be harmful in critically ill patients in whom concomitant infection, systemic inflammation, and pre-existing tissue hypoxia could augment susceptibility to oxygen toxicity [17–19]. These concerns have been heightened by observational studies suggesting that clinicians frequently administer unneeded low but still supraphysiologic FiO_2_ levels in intensive care unit (ICU) patients [20, 21]. Clinical studies aiming to define acceptable low oxygen levels in critically ill patients by comparing conservative and liberal oxygen protocols have been at odds. While one systematic review comparing such studies reported that for each percentage point increase in SaO_2_ in the liberal group increased the relative risk of mortality, another review found no such relationship [22, 23].

Overall, the risks of low but supraphysiologic levels of oxygen remain unclear and continue to be studied clinically. The need for this work has been highlighted by the prolonged oxygen administration many patients with SARS-CoV-2 pneumonia have required [24, 25]. We were, therefore, surprised to find in an informal literature review that in contrast to the many preclinical studies of high FiO_2_ levels, there appeared to be few such studies examining the risks of low but supraphysiologic FiO_2_s. To comprehensively explore this literature, we performed a systematic review of in vivo studies in adult animal models that compared the effects of normobaric oxygen administration with FiO_2_s of ≤ 0.60 and > 0.21 (termed FiO_2_s ≤ 0.60/ > 0.21 below) vs. FiO_2_ = 0.21. Our primary focus was how these FiO_2_s altered outcomes in animals administered infectious or noninfectious inflammatory challenges but studies examining FiO_2_s ≤ 0.60/ > 0.21 alone were also investigated.

## Methods

This systematic review was prepared using the Preferred Reporting Items for Systematic Reviews and Meta-Analyses statement on guidance for literature review and data extraction (Additional file 2). It was registered with the International Prospective Register of Systematic Reviews on 12/3/2021 (PROSPERO-2021-CRD42021285138).

### Literature search and study inclusion

Using search terms and strategies listed in Additional file 3, three authors (S.M., R.D., P.Q.E.) identified relevant studies published in the following databases from inception through 9/30/21: PubMed, EMBASE, and Web of Science. Recovered reports were reviewed for additional references. Studies were included for analysis if they provided data that compared the effects of exposure to FiO_2_s ≤ 0.60/ > 0.21 vs. an FiO_2_ = 0.21 for ≥ 24 h in adult in vivo animal models that either included an infectious or noninfectious inflammatory nonoxygen challenge or did not. Inflammatory challenges were defined as infectious if they included a live microbial agent or noninfectious if the challenge was nonliving (e.g., lipopolysaccharide) but typically associated with an inflammatory response. Inflammatory challenges were ones administered either into the lung, peritoneum or blood. Hyperbaric oxygen, neonatal/infant oxygen, and bronchopulmonary dysplasia studies and non-English publications were not included.

### Data extraction

Three investigators (S.M., R.D, P.Q.E) independently extracted available data from reports using a standardized extraction form (Additional file 4). These data included: country and year of publication; species, strain, age and weight of animals; level, timing and duration of oxygen exposure; type, dose, route and timing of any non-oxygen inflammatory challenge; and number of animals per study-group. Data for survival and measures of lung or non-pulmonary organ injury and lung or systemic levels of immune response parameters were extracted from studies when presented and compared between study groups. When numbers or percentages of animals living or dead were not reported in studies presenting survival curves, authors of reports were contacted to obtain these data. If these data were still not available, animal numbers were calculated from presented survival curves and the total numbers of animals reportedly studied. For all other data, reported mean and median data with variances and/or levels of significance for differences in measures between study groups were recorded. If data were provided in figures alone, means or medians with variances were determined from the figures, and reported significance levels for group comparisons were recorded. To provide representative findings from exposure to higher FiO_2_s, similar data from groups exposed to FiO_2_s > 0.60 in included studies were also recorded.

### Quality of evidence

Two reviewers (S.M and P.Q.E) independently assessed included studies for quality of evidence using a modified version of the Systemic Review Centre for Laboratory Animal Experimentation (SYRCLE) grading system [26, 27]. Studies were examined to determine if the following information was provided: a primary outcome; sample size or power calculation; randomization of challenges; confirmation of baseline similarity of study groups (e.g., age, weight); blinding of challenges and outcome assessments; and randomized animal housing.

### Statistical methods

For mortality, we used the odds ratios of survival to compare groups (FiO_2_ ≤ 0.60/ > 0.21 or FiO_2_ > 0.60 vs. FiO_2_ = 0.21). Continuous outcomes (e.g., body weight, measures of lung injury, etc.) were analyzed using standardized mean difference (SMD). Studies were combined using a random-effect models [28]. In retrieved studies in which more than one group with an increased FiO_2_ regimen (e.g., FiO_2_ = 0.40 and 0.60) was compared to a common FiO_2_ = 0.21 control group, if the survival results across these groups had heterogeneity (*I*^2^) with significance levels *p* ≥ 0.10, these results were pooled (using random-effect models) to provide a single survival effect for the study. If results from groups within studies differed with a *p* < 0.10, these groups were included individually in analysis. The effects of increased FiO_2_’s was then examined across studies employing the same animal type and then across different animal types. Influence of duration of oxygen exposure on lung injury parameters was assessed in meta-regression if there were ≥ 5 studies and/or groups available for analysis. Heterogeneity among studies was assessed using the *Q* statistic and *I*^2^ value. [29]. All analyses were performed using R [30](version 4.2.0) packages *meta* (version 5.2–0). [31]. Two-sided *p* values ≤ 0.05 were considered significant.

## Results

Of 14,369 retrieved reports and after review of references, 24 studies met inclusion criteria (Additional file 1: Fig. S1) [32–55]. Ten studies compared the effects of FiO_2_s ≤ 0.60/ > 0.21 to FiO_2_ = 0.21 in animals challenged with an infectious (*n* = 6 studies) or noninfectious (*n* = 4) inflammatory challenge [32–34, 37, 38, 43, 45, 49, 51, 52] and 14 studies compared these FiO_2_s alone [35, 36, 39–42, 44, 46–48, 50, 53–55]. The two groups of studies were examined separately. More than 600 reports investigating only FiO_2_s > 0.60 were excluded.

### Studies with an infectious or noninfectious inflammatory challenge

Table 1 summarizes the characteristics of the 10 studies comparing the effects of FiO_2_s ≤ 0.60/ > 0.21 to an FiO_2_ = 0.21 in animals also administered an inflammatory challenge. The FiO_2_s ≤ 0.60/ > 0.21 investigated included 0.40, 0.50 or 0.60 alone in two, three and two studies, respectively, and both 0.40 and 0.60 in two, or 0.27 and 0.60 in one. Five studies included animals exposed to FiO_2_s > 0.60 (0.70 to 1.0). The longest oxygen exposure periods were 1d in 4 studies, 4d in two studies and 6, 7, 8 and 35d in one study each. No experiment utilized mechanical ventilation. Infectious challenges included cecal ligation and puncture (CLP) in three studies and either intratracheal (IT) *Legionella pneumoniae* (*L. pneumoniae*) or IT *Klebsiella pneumonia* (*K. pneumoniae*) in one each. One study each administered intraperitoneal (IP) lipopolysaccharide (LPS), IT LPS, IT hydrochloric acid (HCL) or intracardiac (IC) oleic acid. Six studies administered the inflammatory challenge immediately before starting oxygen (0 h) and one study each administered it 4, 12, 18 or 48 h before (Table 1). Three studies included control groups exposed to FiO_2_s ≤ 0.60/ > 0.21 that were administered a noninflammatory control challenge. None of these studies examined whether the effects of increased FiO_2_s with or without an inflammatory challenge were additive or synergistic and these groups are not examined further here. Numbers of animals in experimental groups within studies are summarized in Additional file 1: Table S1.

**Table 1:** Summary of O_2_ + nonO_2_ inflammatory challenge study characteristics

Author (year)	Country	Animals				Oxygen regimens		Inflammatory challenge		Measures reported
		Type (Strain)	Sex	Age	Wgt	FiO_2_	Duration	Type	Route and Dose, timing	
Cheney (1980)	US	Dog (Mongrel)	NR	NR	24 ± 4 kg	0.21, 0.50	4 or 8d	Oleic acid	IC; 0.09 ml/kg; − 4 h	Survival; lung injury^a^; systemic and PA hemodynamics
Rinaldo (1985)	US	Rats (SD)	NR	6–8 weeks	180–230 g	0.21, 0.60	1, 3, or 6d	LPS	IP; 2.5, 7.5 mg/kg; 0 h	Survival; lung injury; lung or systemic immune response parameters^b^
Garner (1988)	US	Rats (SD)	NR	NR	240–270 g	0.21, 0.40, 0.80	7d	CLP, sham	0 h	Survival; lung injury^a^
Cantor (1990)	US	Hamster (Syrian Gold)	NR	NR	100 g	0.21, 0.60	4, 7, or 35d	IT elastase, NS	IT; 30U Els in 0.3 ml NS; 0.3 ml NS; 2d, − 48 h	Lung injury; lung or systemic immune response parameters, lung elastin C14 uptake
Knight (2000)	US	Rabbits (NZW)	NR	Adult	2 kg	0.21, 0.50	1d	HCL, no HCL	IT; 2.4 ml/kg; 0 h	Survival; lung injury; surfactant activity
Nara (2004)	Japan	Mice (C57BL6)	F	6–8 weeks	NR	0.21, 0.50, 0.70, 0.90	2 or 4d	*L. pneumo.*, no infection^e^	IT; 5 × 10^6^ CFU/mouse; 0 h	Survival; lung injury; lung or systemic immune response parameters; lung bacteria
Sun (2006)	China	Rats (SD)	M	NR	180-220 g	0.21, 0.40, 1.0	1d	*K. pneumo*, NS^e^	IT; 1.3 × 10^8^ CFU/rat; NS; 0 h	Lung injury; lung or systemic immune response parameters; surfactant activity^c^; blood/lung bacteria
Aggarwal (2010)	US	Mice (C57BL6)	M	6–8 weeks	NR	0.21, 0.27, 0.60	0.5, 2,3 or 4d	LPS, H_2_O	IT; 0.375ug/g; sterile H_2_O; − 12 h	Lung injury; lung or systemic immune responses parameters
Rodriguez-Gonzalez (2014)	Spain, Canada	Rats (SD)	M	13 weeks	257 ± 21 g	0.21, 0.40, 0.60, 1.0	1d	CLP, sham^e^	0 h	Survival; lung or systemic immune response parameters; blood, lung, urine, meningeal bacteria;
Garcia-Laorden (2020)	Spain, Can	Rats (SD)	M	12–13 weeks	285 ± 21 g	0.21, 0.40, 0.60, 1.0	1d	CLP, sham, healthy	− 18 h	Survival; lung injury; lung or systemic immune response parameters; serum organ injury markers^d^

*BAL* bronchoalveolar lavage, *CLP* cecal ligation and puncture, *Els* elastase, *F* female, *HCL* hydrochloric acid, *IC* intracardiac, *IT* intratracheal, *K. pneumo.* *Klebsiella pneumophilia*, *L. pneumo* *Legionella pneumophilia*, *LPS* lipopolysaccharide, *M* male, *NR* not reported, *NS* normal saline, *NZW* New Zealand White, *PA* pulmonary artery, *ROS* reactive oxygen species, *SD* Sprague–Dawley, *Sham* sham CLP, *U* units, *UC* unclear, *Wgt* weight, *Wks* weeks; 0 h, − 4 h, − 12 h, − 18 h—challenge administered at the time of or 4, 12 or 18 h before O2 therapy provided, respectively

^a^Lung injury measures included one or more of the following: arterial oxygen pressure; lung weights, lung wet to dry weight ratios; bronchoalveolar lavage (BAL) volume, cell, protein or albumin concentrations; alveolar permeability to solute; lung pressure/volume relationships, lung volumes, diffusion capacity; lung histologic changes; and/or Type-2 cell dysfunction

^b^Lung or systemic immune response parameters included one or more of the following: BAL cellularity and polymorphonuclear neutrophil cell numbers; BAL or lung tissue macrophages; BAL, lung tissue or serum cytokines, inducible nitric oxide synthase, nuclear transcription factors, apoptosis markers, S100b, reactive oxygen species, and/or myeloperoxidase measures

^c^Surfactant activity measures included: BAL Type 2 cell phosphatidyl choline uptake, total phospholipids (TPL), desaturated phosphatidylcholine (DSPC), DSPC/TPL, and/or surface tension

^d^Serum organ injury markers included: blood–urea–nitrogen, creatinine, aspartate or alanine aminotransferases, alkaline phosphatase, lactate dehydrogenase, troponin, serum neuron specific enolase, and/or creatine phosphokinase

^e^Control challenge only administered with FiO_2_ = 0.21

**Fig. 1:**
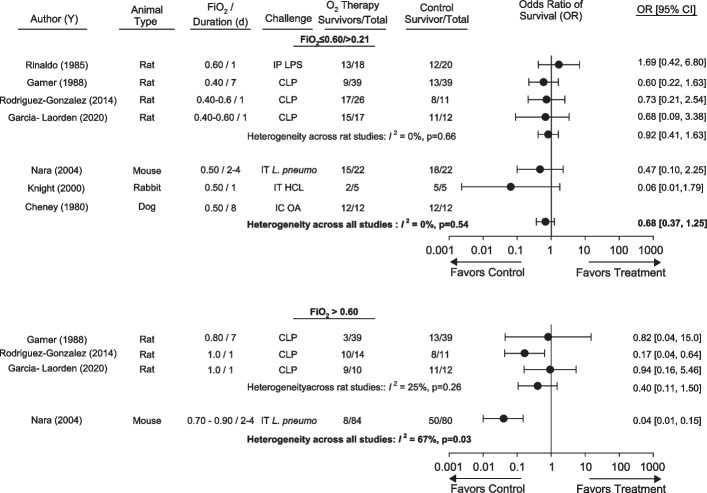
Effects of either FiO_2_s ≤ 0.60 and > O.21 (FiO_2_ ≤ 0.60/ > O.21) (upper panel) or FiO_2_s ≥ 0.60 (lower panel) vs. FiO_2_s = 0.21 (controls) on the odds ratios of survival (95%CIs) (OR) in studies [author (y)] that also administered an infectious or noninfectious inflammatory challenge in animals. Animal type, increased FiO_2_ level and duration, and inflammatory challenge route and type employed in studies are also shown. Also noted are the numbers of animals surviving and total numbers of animals in oxygen or control groups. Data from individual regimens of oxygen or challenge that were pooled within studies based on nonsignificant heterogeneity (*I*^2^ level of significance, *p* ≥ 0.10) comparing the regimens are shown in Additional file 1: Fig. S2. Overall ORs were pooled across animal type and studies if the significance level for heterogeneity was *p* ≥ 0.10. While an overall OR could be calculated for FiO_2_ ≤ 0.60/ > O.21 (*I*^2^ = 0%, *p* = 0.54), this was not possible for FiO_2_s ≥ 0.60 due to high heterogeneity (*I*^2^ = 67%, *p* = 0.03) comparing rat and mouse. *IP* intraperitoneal, *CLP* cecal ligation and puncture, *IT* intratracheal, *IC* intracardiac, *L. p.* *Legionella pneumoniae*, *HCL* hydrochloric acid, *OA* oleic acid

Seven studies compared the effects of FiO_2_s ≤ 0.60/ > 0.21 vs. FiO_2_ = 0.21 on survival in animals administered an inflammatory challenge (Table 1, Fig. 1, Additional file 1: Fig. S2). Survival results for two studies were determined from the average of the range of animals studied and the survival curves provided (see methods). Four studies were conducted in rats, and one each in mice, rabbits or dogs. Two studies examined two FiO_2_s ≤ 0.60/ > 0.21, one examined the same FiO_2_ administered for two different time periods, and one examined the same oxygen regimen with two different LPS doses. For studies that examined two FiO_2_s ≤ 0.60/ > 0.21 or two LPS challenges, there was low heterogeneity across groups within each of these studies, (*I*^2^ ≤ 14%, *p* ≥ 0.28, Additional file 1: Fig. S2) and these groups were, therefore, pooled within studies for analysis (see methods). Across animal types studied, compared to FiO_2_ = 0.21, FiO_2_s ≤ 0.60/ > 0.21 had consistent effects on the odds ratio of survival (95%CI) (OR) that overall was on the side of harm but was not significant [0.68 (0.38, 1.23), *p* = 0.21; *I*^2^ = 0%, *p* = 0.57] (Fig. 1). Notably, in four studies oxygen was administered for only 1d and in one study for only 2 to 4d (Fig. 1). Four studies reported survival in animals administered FiO_2_s > 0.60. In three of these studies in rats, oxygen exposure had effects on the OR on the side of harm but which was not significant [0.40 (0.11,1.50), *p* = 0.18; *I*^2^ = 25%, *p* = 0.26)]. Across eight groups investigated in the remaining study in mice, FiO_2_s > 0.60 decreased the overall OR [0.04 (0.01, 0.15), *p* < 0.0001; *I*^2^ = 34%, *p* = 0.18] (Additional file 1: Fig. S2). Significant heterogeneity prevented combining these rat and mouse results for studies with FiO_2_s > 0.60 (*I*^2^ = 67%, *p* = 0.03) (Fig. 1).

**Fig. 2:**
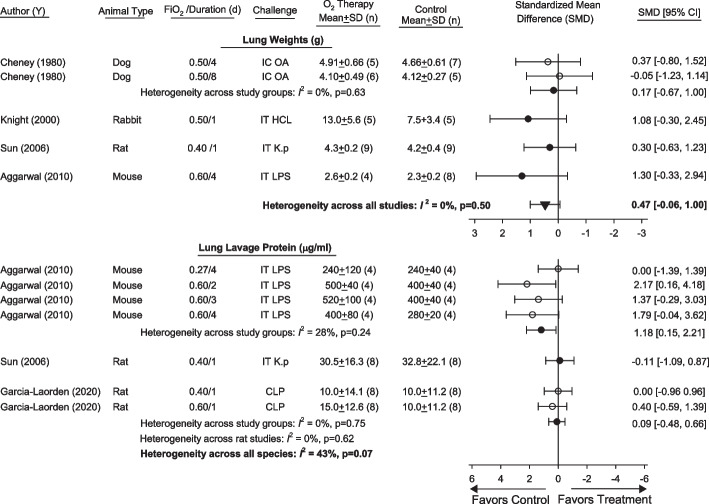
Effects of FiO_2_sT ≤ 0.60 and > O.21 (FiO_2_ ≤ 0.60/ > O.21) vs. FiO_2_s = 0.21 (controls) on standardized mean differences (± SD) (SMD) in lung weights and lung lavage protein in studies [author (y)] that also administered animals an infectious or noninfectious inflammatory challenge. Animal type, FiO_2_ level and duration, inflammatory challenge route and type employed in studies are shown. Data from studies used to calculate SMDs for these parameters and parameter units are shown in Additional file 1: Table S2. Open circles represent results from individual regimens of oxygen or challenge within studies examining more than one regimen, that could be pooled (*I*^2^ level of significance, *p* ≥ 0.10) to report an overall SMD for the study, shown by solid circles. Results shown by solid circles were then used to determine whether SMDs could be pooled across studies in the same species and then across all studies. Also shown are the number of animals (*n*) in study groups. While an overall SMD could be calculated across studies for lung weights (*I*^2^ = 0%, *p* = 0.50; inverted triangle), differences between mouse and rat studies prevented this for lung protein (*I*^2^ = 43%, *p* = 0.07). *CLP* cecal ligation and puncture, *IT* intratracheal, *IC* intracardiac, *K. p.* *Klebsiella pneumoniae*, *HCL* hydrochloric acid, *LPS* lipopolysaccharide, *OA* oleic acid

**Fig. 3:**
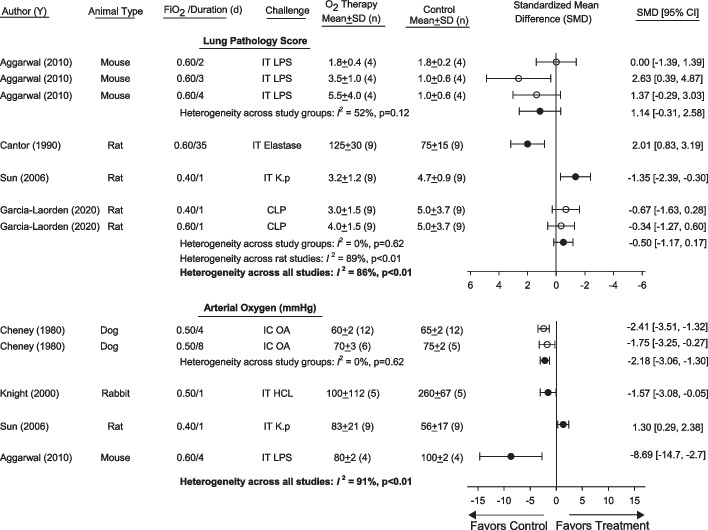
Effects of FiO_2_s ≤ 0.60 and > O.21 (FiO_2_ ≤ 0.60/ > O.21) vs. FiO_2_s = 0.21 (controls) on standardized mean differences (± SD) (SMD) in pathology scores and arterial oxygen levels in studies [author (y)] that also administered animals an infectious or noninfectious inflammatory challenge. Animal type, FiO_2_ level and duration, and inflammatory challenge route and type employed in studies are shown. Data from studies used to calculate SMDs for these parameters and parameter units are shown in Additional file 1: Table S2. Open circles represent results from individual regimens of oxygen or challenge within studies examining more than one regimen, that could be pooled (*I*^2^ level of significance, *p* ≥ 0.10) to report an overall SMD for the study, shown by solid circles. Results shown by the solid circles were then used to determine whether SMDs could be pooled across studies in the same species and then across all studies. Also shown are the number of animals (*n*) in study groups. Differences across studies for lung pathology scores (*I*^2^ = 86%, *p* < 0.01) and across species for arterial oxygen levels (*I*^2^ = 91%, *p* < 0.01) prevented estimation of overall SMDs for either parameter. *CLP* cecal ligation and puncture, *IT* intratracheal, *IC* intracardiac, *K. p.* *Klebsiella pneumoniae*, *HCL* hydrochloric acid, *LPS* lipopolysaccharide, *OA* oleic acid

Three or four studies compared the effects of one or more regimen of FiO_2_s ≤ 0.60/ > 0.21 vs. FiO_2_ = 0.21 on lung injury measures including lung weights, lavage protein concentrations, pathology scores and/or arterial oxygen pressures. The animal types, oxygen regimens, inflammatory challenges and the measures, variances and units provided in studies are presented in Additional file 1: Table S2. These data were used to compare standardized mean differences (95%CI) (SMD) in these measures within studies and across animal types (Figs. 2, 3). Compared to FiO_2_ = 0.21, FiO_2_ ≤ 0.60/ > 0.21 increased SMD in lung weights across four studies in trends approaching significance and with low heterogeneity [0.47 (− 0.07,1.00), *p* = 0.09; *I*^2^ = 0%, *p* = 0.50). One study in mice reported that FiO_2_ = 0.50 for 4d in mice challenged with IT *L. pneumophilia* significantly increased lung weight but was not analyzed, because the type of variance was not identified (Additional file 1: Table S2).[45]. Differences in the effects of FiO_2_ ≤ 0.60/ > 0.21 between the species studied for lung lavage protein (mouse vs. rat, *I*^2^ = 43%, *p* = 0.17) and arterial oxygen measures (mouse vs. rat vs. rabbit vs. dog, *I*^2^ = 91%, *p* < 0.01) prevented estimation of overall SMDs (Figs. 2, 3). Notably, two studies in which FiO_2_ ≤ 0.60/ > 0.21 had effects on oxygen measures on the side of harm (i.e., favoring control) may have reported arterial oxygen measures in animals receiving some level of oxygen support thereby blunting the possible adverse effects of these FiO_2_s [34, 43]. While overall lung pathology scores did not differ comparing species (*p* = 0.36), significant heterogeneity across studies (*I*^2^ = 86%, *p* < 0.01) also prevented estimation of an overall SMD (Fig. 3). For parameters with more than five studies for analysis, meta-regressions analysis did not show a strong relationship [slope (± SE)] between duration of oxygen exposure and the effects of FiO_2_ ≤ 0.60/ > 0.21 on lung pathology scores [0.067 (± 0.035), *p* = 0.06; residual heterogeneity *I*^2^ = 67%] and showed no relationship with oxygenation [*p* = 0.49 for the slope; residual heterogeneity *I*^2^ = 92%].

The effects of FiO_2_ ≤ 0.60/ > 0.21 on measures of lung injury reported on in only one or two studies were not analyzed but are presented in Additional file 1: Table S2. Measures of lung injury with FiO_2_s > 0.60 were only reported on in one or two studies (Additional file 1: Table S3).

Lung or systemic immune response measures were reported with one or more regimen of FiO_2_ > 0.60 and an inflammatory challenge in three studies and with FiO_2_s ≤ 0.60/ > 0.21 in six studies and are presented in Additional file 1: Tables S3 and S4, respectively. Except for one study in which polymorphonuclear cell depletion reduced oxygen induced lung injury with IT LPS challenge [32], it is difficult to determine how changes in the various immune response measures reported in these studies contributed to the effects of increased FiO_2_s on outcomes.

### Studies without an inflammatory challenge

Table 2 summarizes characteristics of the 14 studies comparing the effects of FiO_2_ ≤ 0.60/ > 0.21 to an FiO_2_ = 0.21 and not combined with an inflammatory challenge. The FiO_2_s ≤ 0.60/ > 0.21 investigated included 0.60 alone in ten, 0.50 alone in three, and 0.30, 0.40, 0.50 and 0.60 in one. The longest oxygen exposure period investigated was 3d in two studies, 7d in three studies, 21d in two studies and 2.5, 3.75, 8, 14, 42 or 90d in one study each. Ten studies included animals exposed to FiO_2_s > 0.60 (0.65 to 1.0). One study [40] utilized mechanical ventilation, but only after 1w of oxygen exposure and for the purpose of conducting mechanical studies. Numbers of animals in experimental groups within studies are summarized in Additional file 1: Table S5.

**Table 2:** Summary of O_2_ only study characteristics

Author (y)	Country	Animals studied				Oxygen studied		Measures reported
		Type (Strain)	Sex	Age	Wgt	FiO_2_	Duration	
Hackney (1975)	US	Monkey (Squirrel)	M	NR	NR	0.21, 0.60, 0.80	2, 4, 8d	Lung injury^a^, lung or systemic immune response parameters^b^
Hayatdavoudi (1981)	US	Rat (Charles River – CD)	M	NR	300–350 g	0.21, 0.60, 0.85	7d	Body weights; lung injury; lung or systemic immune response parameters; survival with FiO_2_ = 1.0 after prior lower FiO_2_ exposure
Rister (1983)	Germany	Guinea pigs	NR	NR	NR	0.21, 0.30, 0.40, 0.50, 0.60, 0.70, 0.80	0, 18, 42, 60, 90 h	Lung injury
Coursin (1987)	US	Rats (SD)	M	NR	160–200 g	0.21, 0.50, 0.65, 0.80	7, 14, 21, 28, 42d	Body weights; lung injury; lung or systemic immune response parameters; Survival with FiO_2_ = 1.0 after prior lower FiO_2_ exposure
Holm (1987)	US	Rabbits (NZW)	M	NR	1.9–2.2 kg	0.21, 0.60	21d	Lung injury; lung or systemic immune response parameters
Nickerson (1990)	US	Rabbits (NZW)	NR	NR	2.0–3.0 kg	0.21, 0.60	21d	Lung injury
Nylen (1993)	US	Hamsters (Syrian Gold)	M	6weeks	NR	0.21, 0.60	7, 21, 90d	Body weight; lung injury
Van Klaveren (1997)	Belgium, UK	Rats (Wistar)	M	NR	NR	0.21, 0.60, 0.85	7d	Body weight; lung injury; lung or systemic immune response parameters; survival
Belik (2003)	Canada	Rats (SD)	F	NR	250–275 g	0.21, 0.60	14d	Lung injury
Nelin (2003)	US	Rats (SD)	M	NR	250–325 g	0.21, 0.50, 0.90	2.5d	Body weight; lung injury; salicylate conversion to 2,3-DHBA to assess lung hydroxyl radical oxidant production
Hesse (2004)	Germany	Mice (C57BL6/J)	M	12–16 wk	26–27 g	0.21, 0.60, > 0.95	3d	Body weight; lung injury; lung or systemic immune response parameters; body weight; survival
Gan (2011)	US	Rats (SD)	M	NR	275–325 g	0.21, 0.60, 0.85	7d	Body weight; lung injury; lung or systemic immune response parameters
Audi (2012)	US	Rats (SD)	M	NR	300–325 g	0.21, 0.60, 0.95	60–7d 95–2d	Lung injury; lung or systemic immune response parameters
Lagishetty (2014)	US	Mice (C57BL/6 J)	M	9 weeks	NR	0.21, 0.50, 0.75, 1.0	3d	Lung injury; lung or systemic immune response parameters; lung tissue expression of 6 CLOCK proteins (CLOCK, Bmal1, Cry1, Cry2, Per1, Per2)

*F* female, *M* male, *MAC* macrophage, *NR* not reported, *NZW* New Zealand White, *SD* Sprague–Dawley, *UC* unclear, *US* United States, *UK* United Kingdom, *Wgt* weight, *Bmal1—CLOCK* circadian locomotor output cycles kaput, *2,3-DHBA* dihydroxy benzoic acid, *GSH* glutathione, *HMPAQ* Tc labele3d hexamethylpropyleneamine oxide (trapped in the lung by GSH), *DEM* GSH depleter diethyl maleate, *GSH-glutathione* GSSG-oxidized glutathione

^a^Lung injury measures included one or more of the following: arterial oxygen pressure; lung weights, lung wet to dry weight ratios, lung to body weight ratios; bronchoalveolar lavage (BAL) volume, cell, protein or albumin concentrations; alveolar permeability to solute; lung pressure/volume relationships, lung volumes, diffusion capacity; lung histologic changes; pulmonary artery and airway contractile and relaxation function, and/or alveolar macrophage and neutrophil microtubule and microfilament integrity, BAL type-2 cell dysfunction or phospholipid content

^b^Lung or systemic immune response parameters included one or more of the following: BAL cellularity and polymorphonuclear neutrophil cell numbers, BAL or lung tissue macrophages, BAL or lung tissue lymphocytes; BAL or serum cytokine, nitric oxide, or nitric oxide synthase levels; lung antioxidant activity which included HMPAQ retention with or without DEM based on lung to background ratio, lung or BAL glutathione peroxidase (GPx), glutathione (GSH), oxidized glutathione (GSSG), gamma-glutamyltransferase, non-protein-sulfhydral (NPSH), and/or superoxide dismutase (SOD) levels

Two studies [31, 36] compared survival time in rats exposed to FiO_2_ = 1.0 immediately after animals had previously been exposed to either FiO_2_ = 0.21or 0.60 for 7d or FiO_2_ = 0.21 or 0.50 for 42d. Compared to FiO2 = 0.21, survival time was reduced after prior exposure to FiO_2_ = 0.60 [median survival time 66 h vs. 48 h, respectively (no IQRs reported, *n* = 40 animals per group), *p* < 0.01 as reported)] or FiO_2_ = 0.50 (mean ± SD survival time, 67.2 ± 1.1 h vs. 55.5 ± 3.3 h, *p* < 0.001 as reported). One study each in rats or mice noted that all animals survived after exposure to FiO_2_ = 0.60 for 7d or 3d, respectively [41, 53].

**Fig. 4:**
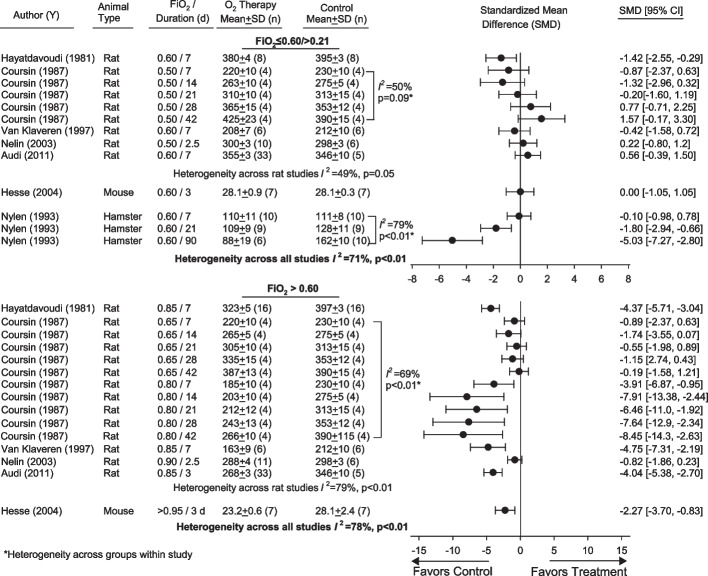
Effects of FiO_2_s ≤ 0.60 and > O.21 (FiO_2_ ≤ 0.60/ > O.21) (upper panel) or FiO_2_ > 0.60 (lower panel) vs. FiO_2_s = 0.21 (controls) on standardized mean differences (± SD) (SMD) in body weights in grams in studies [author (y)] that did not include an infectious or noninfectious inflammatory challenge. Animal type, increased FiO_2_ level, and duration employed in studies or groups are shown. Also shown for each study or group are the standardized means (± SD) and numbers of animals (*n*) used to calculate individual SMDs. Data from studies used to calculate SMDs for these parameters and parameter units are shown in Additional file 1: Table S6. Analysis for two studies (Coursin and Nylen) that included more than one regimen of FiO_2_ ≤ 0.60/ > O.21 or FiO_2_ > 0.60 showed that the levels of significance for heterogeneity (*I*^2^ ≥ 50%, *p* ≤ 0.09) across these regimens prevented pooling groups and these groups are shown as solid circles and used in analysis individually here. Heterogeneity across studies and groups also prevented calculating overall SMDs for FiO_2_ ≤ 0.60/ > O.21 (*I*^2^ = 71%, *p* < 0.01) and FiO_2_ > 0.60 (*I*^2^ = 78%, *p* < 0.01)

Five studies in rats and one each in mice and hamsters, provided data that could be used to compare SMDs in body weight for FiO_2_s ≤ 0.60/ > 0.21 vs. FiO_2_ = 0.21 (Fig. 4, Additional file 1: Table S6). There was substantial heterogeneity across five groups receiving increasingly longer regimens of oxygen in one rat study (*I*^2^ = 50%, *p* = 0.09) and three groups in the hamster study (*I*^2^ = 79%, *p* < 0.01) and these groups could not be pooled within each study, Although FiO_2_s ≤ 0.60/0.21 did not increase the SMD for body weight significantly in any study or group but decreased it significantly in one study and in two groups in another study, there was significant heterogeneity in these SMDs across studies (*I*^2^ = 71%, *p* < 0.01). Meta-regression showed a negative relationship [slope (SE)] between duration of oxygen exposure and body weight [− 0.036 (± 0.016), *p* = 0.03] but residual heterogeneity was high (*I*^2^ = 70%). Five studies in rats and one in mice provided body weight data in animals administered FiO_2_ > 0.60 (Fig. 4, Additional file 1: Table S6). One rat study examined two different FiO_2_s, each for five different time periods, but the results of these 10 groups could not be pooled (*I*^2^ = 69%, *p* < 0.01). While FiO_2_ > 0.60 decreased the SMD in body weight significantly in 9 individual studies or groups and did not increase it in any, there was significant heterogeneity in its overall effects (*I*^2^ = 78%, *p* < 0.01). There was no significant relationship between duration of exposure to FiO_2_ > 0.60 and body weight [0.007 (± 0.048), *p* = 0.89; residual heterogeneity *I*^2^ = 82%].

**Fig. 5:**
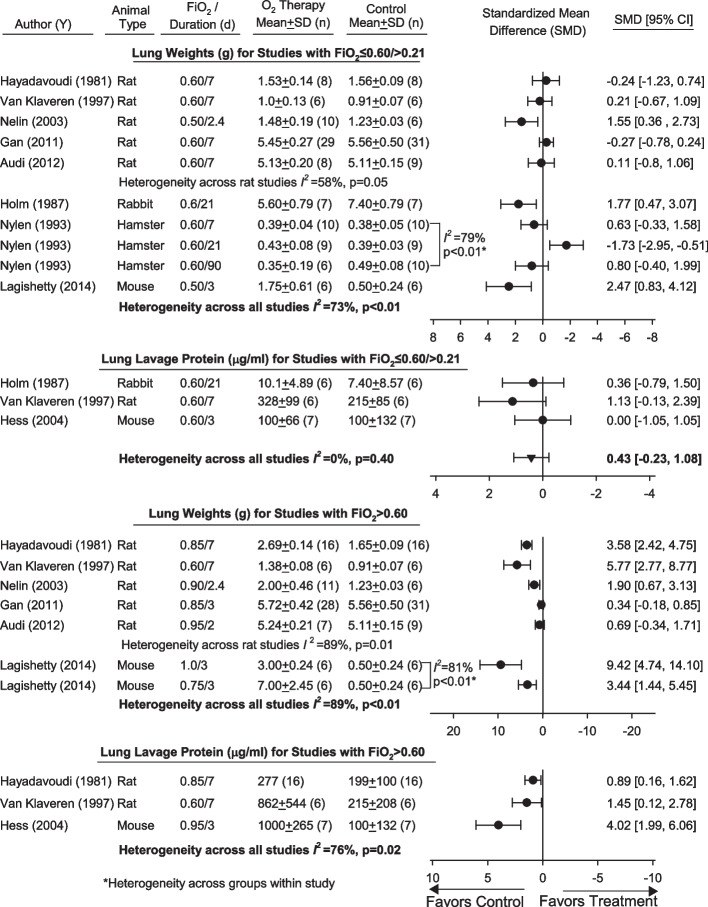
Effects of FiO_2_s ≤ 0.60 and > O.21 (FiO_2_ ≤ 0.60/ > O.21) (Panel **A**) or FiO_2_s > 0.60 (Panel **B**) vs. FiO_2_s = 0.21 (controls) on standardized mean differences (± SD) (SMD) in two measures of lung injury, lung weight and lung lavage protein concentration, in studies [author (y)] that did not include an infectious or noninfectious inflammatory challenge. Animal type and increased FiO_2_ level and duration employed in studies and groups are shown. Also shown for each study or group are the standardized means (± SD) and numbers of animals (*n*) used to calculate individual SMDs. Data from studies used to calculate SMDs for these parameters and parameter units are shown in Additional file 1: Table S7. Analysis for two studies (Nylen and Lagishetty) that included more than one regimen of FiO_2_ ≤ 0.60/ > O.21 or FiO_2_ > 0.60 showed that the level of significance for heterogeneity across these regimens prevented pooling groups (*I*^2^ ≥ 79%, *p* < 0.01; *I*^2^ ≥ 81%, *p* < 0.01) and these are shown as solid circles and used in analysis individually here. While it was possible to calculate an overall SMD for lavage protein concentration in studies with FiO_2_ ≤ 0.60/ > 0.21 (*I*^2^ = 0%, *p* = 0.40), heterogeneity prevented an overall calculation for lung weights for FiO_2_ ≤ 0.60/ > 0.21 (*I*^2^ = 73%, *p* < 0.01) or for lung weights and lavage protein concentration for FiO_2_ > 0.60 (*I*^2^ = 89%, *p* < 0.01 and *I*^2^ = 76%, *p* = 0.02, respectively)

Eight and three studies compared the effects of one or more regimen FiO_2_ ≤ 0.60/ > 0.21 vs. FiO_2_ = 0.21 on measures of lung weight and lavage protein concentrations, respectively (Fig. 5, Additional file 1: Table S7). For lung weights, there was substantial heterogeneity across three groups in one study with hamsters (*I*^2^ = 79%, *p* < 0.01). Although overall lung weights tended to be increased with FiO_2_ ≤ 0.60/ > 0.21, heterogeneity across studies and groups was high and significant (*I*^2^ = 73%, *p* < 0.01). Meta-regression did not show a significant relationship between oxygen exposure time and lung weight [− 0.027 (± 0.012), *p* = 0.03; residual heterogeneity *I*^2^ = 70%]. However, for lavage protein, the effects of FiO_2_ ≤ 0.60/ > 0.21 were consistent across studies and on the side of being increased [0.43 (− 0.23, 1.09), *p* = 0.20; *I*^2^ = 0%, *p* = 0.40)]. The effects of FiO_2_ ≤ 0.60/ > 0.21 on other potential measures of lung injury but only reported in one or two studies and not analyzed further are presented in (Additional file 1: Table S7). Two studies reported electron microscopic changes with FiO_2_ ≤ 0.60/ > 0.21 that were difficult to summarize as to an overall effect [40, 47]. Six and three studies examined the effects of one or more regimen of FiO_2_ > 0.60 on lung weight and lavage protein levels, respectively (Fig. 5, Additional file 1: Table S8). For lung weights, there was substantial heterogeneity across two groups in a mouse study (*I*^2^ = 81%, *p* = 0.02). Overall, FiO_2_ > 0.60 produced SMDs for lung weight across studies on the side of being increased, but there was heterogeneity across groups and studies (*I*^2^ = 89%, *p* < 0.01). There was no relationship between duration of oxygen exposure and lung weight [0.06 (± 0.49), *p* = 0.90; residual heterogeneity *I*^2^ = 93%]. FiO_2_ > 0.60 increased the SMD for lung lavage protein significantly in all three studies measuring it, but again there was substantial heterogeneity for these effects across studies (*I*^2^ = 76%, *p* = 0.02) (Fig. 5). The effects of FiO_2_ > 0.60 on other lung injury measures reported in only one or two studies are presented in Additional file 1: Table S8.

Lung or systemic immune response measures were reported with FiO_2_s ≤ 0.60/ > 0.21 and with FiO_2_ > 0.60 in 8 studies. These measures are available for review in Additional file 1: Tables S8 and S9.

### Quality of evidence

Most studies examining oxygen combined with an inflammatory challenge or alone reportedly matched animals based on age and/or weight (Additional file 1: Table S10). However, information regarding sample size calculations, group randomization procedures, blinding to results assessment, animal removal and randomized animal housing was unclear in more than half of each type of study.

## Discussion

A large body of evidence from animal studies showed that high FiO_2_s have injurious pulmonary effects and has been an important basis for avoiding these levels clinically when possible [1–8]. The present literature search retrieved more than 600 animal studies investigating only FiO_2_ > 0.60. By contrast, despite ongoing questions regarding the potential risks of lower FiO_2_s in critically ill patients, this search retrieved only 10 studies examining the impact of FiO_2_ ≤ 0.60 and > 0.21 (FiO_2_ ≤ 0.60/ > 0.21) on survival or lung injury in animals administered an infectious or noninfectious inflammatory challenge [32–34, 37, 38, 43, 45, 49, 51, 52] and 14 studies examining these FiO_2_s alone [35, 36, 39–42, 44, 46–48, 50, 53–55]. The differing study designs and parameters measured and sometimes inconsistent effects across the limited numbers of studies examining similar or related parameters provide no firm conclusion as to the overall effects of FiO_2_ ≤ 0.60/ > 0.21 in either group of studies. However, findings here do support concerns raised about the potential adverse effects of FiO_2_s levels that are traditionally considered non-toxic and suggest the types of further animal studies that might help inform clinical use of these FiO_2_s [17–19, 22, 23].

Across seven studies examining FiO_2_ ≤ 0.60/0.21 with an accompanying inflammatory challenge, increased oxygen had effects on survival that, while not significant, were highly consistent and on the side of harm. However, in four of these studies, animals were exposed to oxygen for only 24 h. Overall, 6 of the 10 studies administered oxygen and observed animals following an inflammatory challenge for ≤ 4d and only five included an infectious inflammatory challenge. Despite these short exposure and observation periods, among the four studies examining lung weights, a measure of lung injury, FiO_2_s ≤ 0.60/0.21 also had consistent effects that were on the sides of harm. A fifth study that could not be used in analysis reported a similar result [35].

The 14 studies examining FiO_2_s ≤ 0.60/0.21 without an accompanying inflammatory challenge are less informative since clinically relevant questions relate largely to whether lower FiO_2_s aggravate existing lung injury. Notably though, exposure to FiO_2_s = 0.50 and 0.60 alone for 7 days in two studies did decrease survival when animals were subsequently exposed to FiO_2_ = 1.0. While lung lavage protein concentrations were consistent and on the side of harm in the three studies measuring them, body weight and lung weight changes with FiO_2_ ≤ 0.60/0.21 were inconsistent across studies. Consistent with the impact animal studies have had on the avoidance of FiO_2_s > 0.60 clinically, even in the small group of studies investigated here the effects of FiO_2_s > 0.60 on body and lung weights and lavage protein concentrations while variable, were well on the side of harm.

Taken together though, these two groups of studies support the possibility that FiO_2_ ≤ 0.60/0.21 can have harmful effects. When combined with another inflammatory challenge, these effects were apparent with as little as 24 h of exposure. However, sensitivity to hyperoxia varies across and within species and how these limited findings apply clinically is unclear [18, 22, 23]. However, results from controlled studies in healthy humans [56] and observational studies in patients [57–60] were consistent with findings from animal studies regarding the harmful pulmonary effects of FiO_2_s > 0.60. It is likely that appropriately designed animal studies examining FiO_2_ ≤ 0.60/0.21 would be informative as well.

To maximize the benefit and minimize the risks of oxygen therapy in patients with pneumonia or other pulmonary injuries, support is routinely titrated based on arterial oxygen saturation levels or blood oxygen levels on arterial blood gases. Mechanical ventilation is also often required along with oxygen therapy to prevent morbidity or mortality from ventilatory failure as opposed to hypoxemia. The ideal animal model to examine the impact of FiO_2_ levels would be one that included a nonoxygen inflammatory pulmonary challenge and compared the risks and benefits of oxygen therapy titrated over lower or higher ranges. Such a model would also include mechanical ventilation when necessary to prevent confounding by hypoventilation. Such a study would likely require a large animal model and considerable other resources which few laboratories could provide. However, since oxygen titration is standard clinical practice, animal models investigating the risks and benefits of oxygen therapy must attempt to develop the methods to include this type of titration if these models are going to inform clinical practice.

Short of animal models allowing titration of oxygen and ventilatory support, the present review combined with published clinical experience suggest several ways animal models examining the impact of lower FiO_2_s would be most informative clinically. First, these models should emphasize the type of accompanying pulmonary inflammatory challenge typically seen in patients. For medical intensive care units, this would include either bacterial or viral pneumonia. While two studies here employed a pulmonary infectious challenge (*L. pneumoniae* and *K. pneumoniae*), other bacteria such as *S. pneumoniae*, *S. aureus* and *H. influenza* that commonly require ICU admission and oxygen support should be a consideration. Importantly, despite the prevalence of influenza pneumonia and the rapid rise in ICU admissions for SARS-CoV-2 pneumonia, no model examined the impact of FiO_2_s ≤ 0.60/ > 0.21 on a viral pulmonary challenge. Examination of how lower FiO_2_s effect viral pulmonary pathology, especially coronaviruses-like SARS-CoV-2, appears essential [25]. Second, models should include oxygen exposure periods long enough to simulate those critically ill patients are exposed to. For patients with severe enough lung injury who require noninvasive or invasive mechanical ventilation, observational studies suggest that these periods should be at least 5–7 days [61–63]. Experience in patients with SARS-CoV-2 suggest that these periods should be longer [24]. Third, since several studies presented here suggest that FiO_2_ ≤ 0.60/ > 0.21 alone may cause some level of pulmonary injury, studies examining how potential injury with these FiO_2_s interacts with inflammatory pulmonary challenges, i.e., are these effects additive or synergistic, would be most informative. Such studies would require sufficient subject numbers to test for these interactions. Fourth, consensus and consistent use of measures of lung injury considered most informative in studies examining the effects of oxygen therapy on inflammatory lung injury would allow more reliable analysis across studies. Finally, quality of the studies analyzed here was weak. It was unclear in most cases whether studies included sample size calculations, animal randomization procedures, blinding of study results, removal of animals during oxygen exposure periods and randomized animal housing. Future preclinical studies examining the effects of FiO_2_ ≤ 0.60/ > 0.21 on inflammatory lung injury would be strengthened by providing explicit information about these study design components.

There are potential limitations to this systematic review. First, while our search terms were broad and we included additional reports after reviewing references from studies undergoing full paper review, we may have failed to retrieve all studies meeting our inclusion criteria. Second, sensitivity analysis examining sources of heterogeneity was restricted due to the limited numbers of studies available for review for individual parameters. Furthermore, given the heterogeneity of the models used and the outcomes measured and reported, interpretation and generalizability of these results and analysis are limited. Third, in studies where differing regimens of oxygen could not be pooled for analysis, control groups were employed repetitively. Fourth, for almost all studies analyzed it was not possible to determine how reported changes in immune response measures contributed to outcomes. Finally, while oxygen measures when available were employed as a measure of lung injury, increased FiO_2_s in animals at the time of measurement may have blunted reductions in this parameter making them less informative.

In conclusion, while the potential impact of lower FiO_2_s on lung injury in critically ill patients continues to be a concern, few preclinical studies have addressed this question. Those that have, have been limited in terms of the oxygen exposure periods and types of accompanying inflammatory lung injury studied. Given the impact animals studies have had on recommendations regarding the avoidance of toxic higher FiO_2_s clinically, additional animal studies appear warranted to explore how lower FiO_2_s effect lung injury in patients.

*Take home message* While hyperoxia with FiO_2_ > 0.60 is avoided in the Intensive Care Unit due in large part to animal studies showing harm, less is known about the effects of low but still supraphysiologic (FiO_2_ ≤ 0.60 but > 0.21) oxygen supplementation. This review highlights the need for more well-designed animal models to evaluate the effects of low but still supraphysiologic (FiO_2_ ≤ 0.60 but > 0.21) oxygen supplementation with a concurrent inflammatory insult similar to patients seen in the Intensive Care Unit.

## Supplementary Information

**Additional file 1: Figure S1.** Flow diagram for the literature search. **Figure S2.** Effects of FiO_2_ ≤ 0.60 and > O.21 (FiO_2_ ≤ 0.60/ > O.21) (upper panel) or FiO_2_ ≥ 0.60 (lower panel) vs. FiO_2_s = 0.21 (controls) on the odds ratios of survival (95%CIs) (OR) in studies [author (y)] that also administered an infectious or noninfectious inflammatory challenge in animals. These were studies that included more than one regimen of oxygen or dose of an inflammatory challenge and these groups are shown individually here. Animal type, increased FiO_2_ level and duration, inflammatory challenge route and type employed and the numbers of surviving and total animals challenged in oxygen or control groups are shown. Open circles show ORs for groups within studies and solid circles show the overall OR for a study when groups could be pooled (*I*^2^ level of significance, *p* ≥ 0.10). These pooled ORs were then employed for overall analysis (Fig. 1). *IP* intraperitoneal, *CLP* cecal ligation and puncture, *IT* intratracheal, *L. p.* *Legionella pneumoniae*, *LPS* lipopolysaccharide, *LD* low dose, *HD* high dose. **Table S1.** Animal numbers for O2 + nonO2 inflammatory challenge studies. **Table S2.** Results of lung injury measures reported in O2 + nonO2 inflammatory challenge studies for groups exposed to FiO2s≤0.60 and >0.21 or FiO2=0.21. **Table 3.** Results of lung injury and immune response measures reported in O2 + nonO2 inflammatory challenge studies for groups exposed to FiO2 >0.60 or FiO2=0.21. **Table S4.** Results of immune response measures reported in O2 + nonO2 inflammatory challenge studies for groups exposed to FiO2s≤0.60 and >0.21. **Table S5.** Animal numbers for O2 only studies. **Table S6.** Body weights following oxygen exposure for O2 only studies. **Table S7.** Results of lung injury measures reported in O2 only studies for groups exposed to FiO2s≤0.60 and >0.21 or FiO2=0.21. **Table S8.** Results of immune response measures reported in O2 only studies for groups exposed to FiO2s≤0.60 and >0.21 or=0.21. **Table S9.** Results of lung injury and immune response measures reported in O2 only studies for groups exposed to FiO2s>0.60 or FiO2=0.21. **Table S10.** Quality of evidence, adapted from SYRCLE.

**Additional file 2. **27-item PRISMA Checklist for Systematic Reviews.

**Additional file 3. **Search strategy used for PUBMED, Web of Science, and EMBASE.

**Additional file 4. **Extraction form utilitzed by authors for O2 only studies and O2 with non-O2 studies.

## Data Availability

Not applicable.
