# Supplementary material for: Effect of low-to-moderate hyperoxia on lung injury in preclinical animal models: a systematic review and meta-analysis
Source: Intensive Care Med Exp. 2023 Apr 24;11:22. doi: 10.1186/s40635-023-00501-x (PMC10122981; doi:10.1186/s40635-023-00501-x)
Supplement: Supplementary file 1 — Additional file 1: Figure S1. Flow diagram for the literature search. Figure S2. Effects of FiO2 ≤ 0.60 and > O.21 (FiO2 ≤ 0.60/ > O.21) (upper panel) or FiO2 ≥ 0.60 (lower panel) vs. FiO2s = 0.21 (controls) on the odds ratios of survival (95%CIs) (OR) in studies [author (y)] that also administered an infectious or noninfectious inflammatory challenge in animals. These were studies that included more than one regimen of oxygen or dose of an inflammatory challenge and these groups are shown individually here. Animal type, increased FiO2 level and duration, inflammatory challenge route and type employed and the numbers of surviving and total animals challenged in oxygen or control groups are shown. Open circles show ORs for groups within studies and solid circles show the overall OR for a study when groups could be pooled (I2 level of significance, p ≥ 0.10). These pooled ORs were then employed for overall analysis (Fig. 1). IP intraperitoneal, CLP cecal ligation and puncture, IT intratracheal, L. p. Legionella pneumoniae, LPS lipopolysaccharide, LD low dose, HD high dose. Table S1. Animal numbers for O2 + nonO2 inflammatory challenge studies. Table S2. Results of lung injury measures reported in O2 + nonO2 inflammatory challenge studies for groups exposed to FiO2s≤0.60 and >0.21 or FiO2=0.21. Table 3. Results of lung injury and immune response measures reported in O2 + nonO2 inflammatory challenge studies for groups exposed to FiO2 >0.60 or FiO2=0.21. Table S4. Results of immune response measures reported in O2 + nonO2 inflammatory challenge studies for groups exposed to FiO2s≤0.60 and >0.21. Table S5. Animal numbers for O2 only studies. Table S6. Body weights following oxygen exposure for O2 only studies. Table S7. Results of lung injury measures reported in O2 only studies for groups exposed to FiO2s≤0.60 and >0.21 or FiO2=0.21. Table S8. Results of immune response measures reported in O2 only studies for groups exposed to FiO2s≤0.60 and >0.21 or=0.21. Table S9. Re [file 40635_2023_501_MOESM1_ESM.pdf]

Figure S1

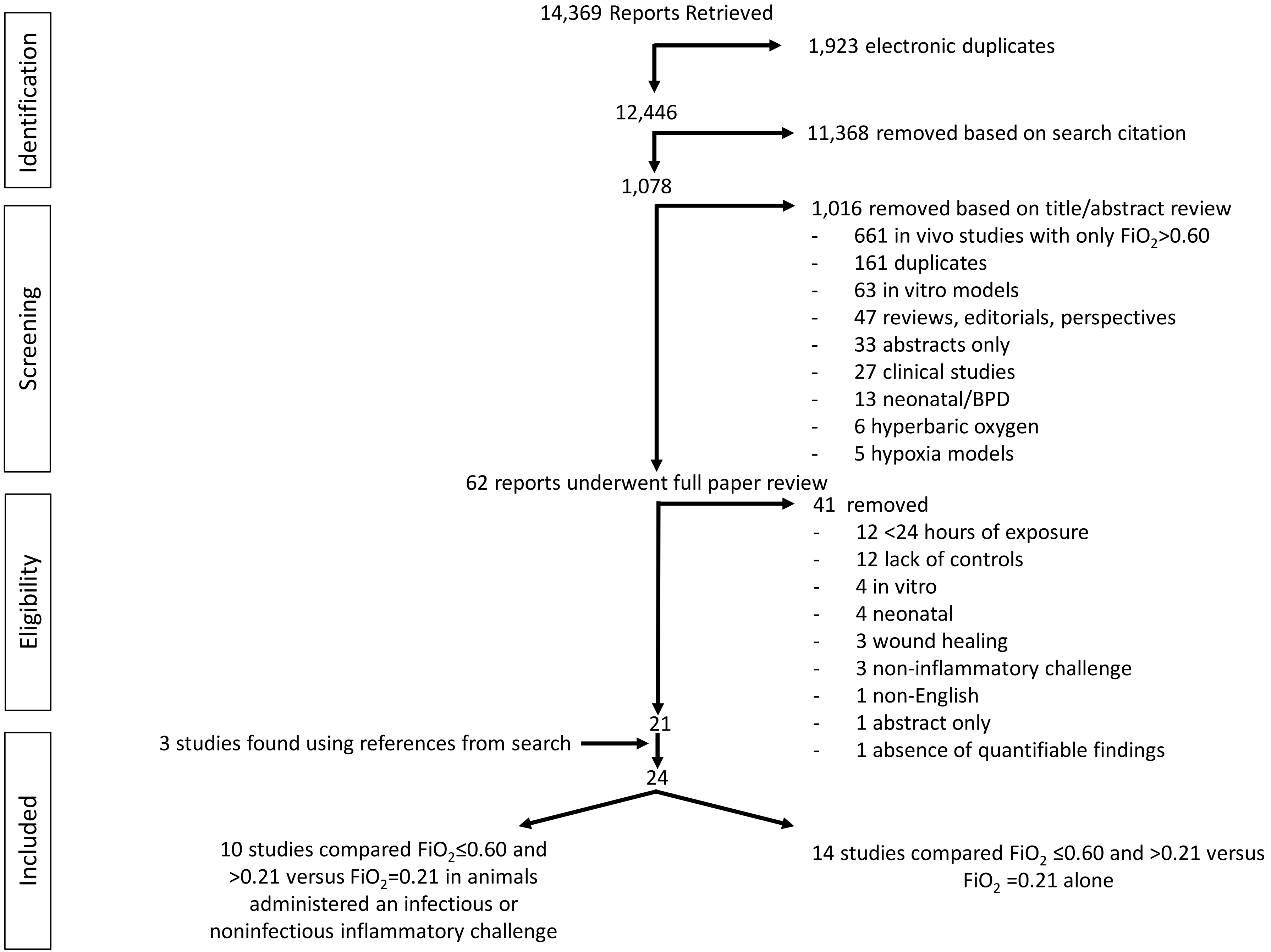
Identification
Screening
Eligibility
Included
14,369 Reports Retrieved
1,923 electronic duplicates
12,446
11,368 removed based on search citation
1,078
1,016 removed based on title/abstract review
- 661 in vivo studies with only FiO_2>0.60
- 161 duplicates
- 63 in vitro models
- 47 reviews, editorials, perspectives
- 33 abstracts only
- 27 clinical studies
- 13 neonatal/BPD
- 6 hyperbaric oxygen
- 5 hypoxia models
62 reports underwent full paper review
41 removed
- 12 <24 hours of exposure
- 12 lack of controls
- 4 in vitro
- 4 neonatal
- 3 wound healing
- 3 non-inflammatory challenge
- 1 non-English
- 1 abstract only
- 1 absence of quantifiable findings
21
3 studies found using references from search
24
10 studies compared FiO_2≤0.60 and >0.21 versus FiO_2=0.21 in animals administered an infectious or noninfectious inflammatory challenge
14 studies compared FiO_2 ≤0.60 and >0.21 versus FiO_2 =0.21 alone

Figure S2

Author (Y)	Animal Type	FiO_2 /Duration (d)	Challenge	O_2 Therapy Survivors/Total	Control Survivor/Total	OR [95% CI]
			**FiO_2≤0.60/>0.21**			
Rinaldo (1985)	Rat	0.60 / 1	LD LPS	8/10	7/10	1.71 [0.22, 13.4]
Rinaldo (1985)	Rat	0.60 / 1	HD LPS	5/8	5/10	1.67 [0.25, 11.1]
**Heterogeneity across groups I^2 = 0%, p=0.98**						**1.69 [0.42, 6.80]**
Nara (2004)	Mouse	0.50 / 2	*L. p.*	10/12	9/11	1.11 [0.13, 9.61]
Nara (2004)	Mouse	0.50 / 4	*L. p.*	5/10	9/11	0.22 [0.03, 1.59]
**Heterogeneity across groups I^2 = 14%, p=0.28**						**0.47 [0.10, 2.25]**
Rodriguez-Gonzalez (2014)	Rat	0.40 / 1	CLP	9/15	8/11	0.56 [0.10, 3.2]
Rodriguez-Gonzalez (2014)	Rat	0.60 / 1	CLP	8/11	8/11	1.00 [0.15, 6.53]
**Heterogeneity across groups I^2 = 0%, p=0.65**						**0.73 [0.21, 2.54]**
Garcia- Laorden (2020)	Rat	0.40 / 1	CLP	7/8	11/12	0.63 [0.03, 11.9]
Garcia- Laorden (2020)	Rat	0.60 / 1	CLP	8/9	11/12	0.73 [0.04, 13.5]
**Heterogeneity across groups I^2 = 0%, p=0.95**						**0.68 [0.09, 5.38]**
			**FiO_2 > 0.60**			
Nara'2004	Mouse	0.90 / 2	*L. p. (5x10^7)*	0/11	0/10	
Nara'2004	Mouse	0.90 / 2	*L. p. (1.6x10^7)*	0/10	6/8	0.02 [0.00, 0.45]
Nara'2004	Mouse	0.90 / 2	*L. p. (5x10^6)*	0/11	12/12	0.01 [0.00, 0.10]
Nara'2004	Mouse	0.90 / 2	*L. p. (1.6x10^6)*	0/10	0/10	
Nara'2004	Mouse	0.70/ 2	*L. p. (5x10^6)*	4/11	8/11	0.21 [0.04, 1.31]
Nara'2004	Mouse	0.70 / 4	*L. p. (5x10^6)*	2/9	8/11	0.11 [0.01, 0.84]
Nara'2004	Mouse	0.90 / 2	*L. p. (5x10^6)*	2/10	8/9	0.03 [0.01, 0.42]
Nara'2004	Mouse	0.90 / 4	*L. p. (5x10^6)*	0/12	8/9	0.01 [0.00, 0.20]
**Heterogeneity across groups I^2 = 34%, p=0.18**						**0.04 [0.01, 0.15]**

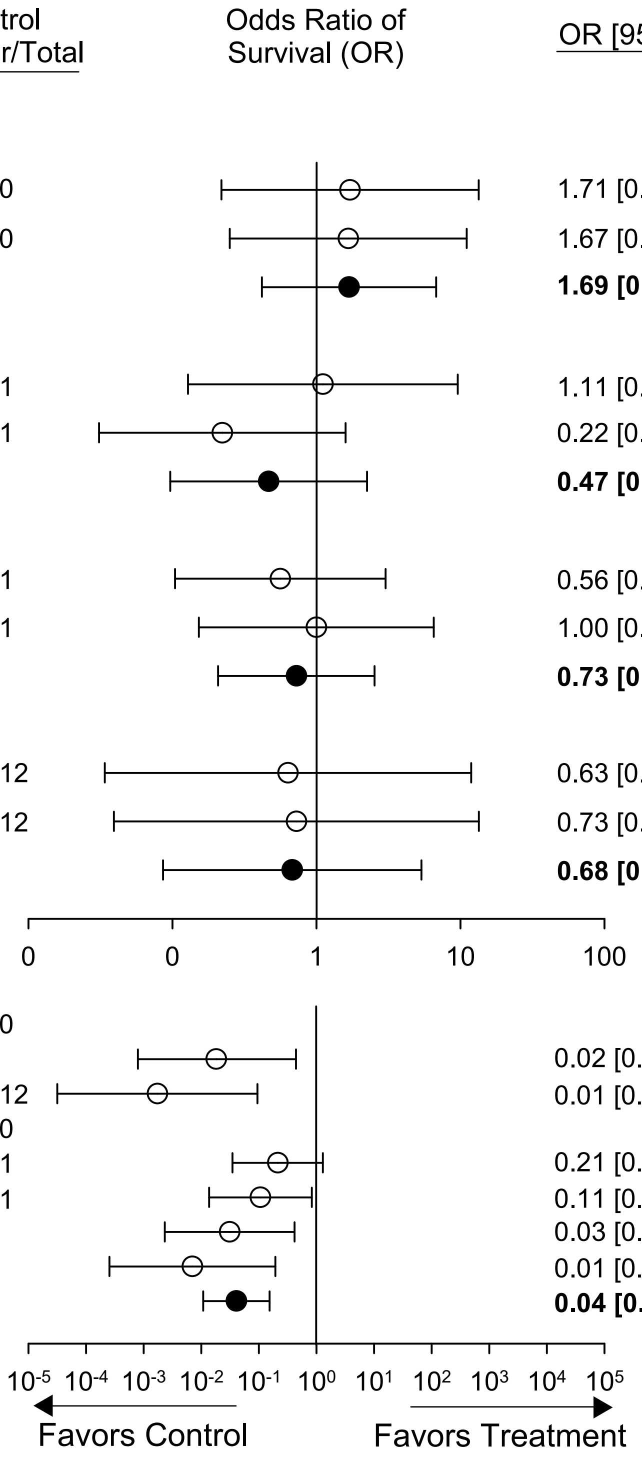

Table S1. Animal numbers for O_2 + nonO_2 inflammatory challenge studies						
Author (y)	Type of Animal Studied	Oxygen Regimens Compared		Inflammatory Challenge Type	Animal Number per Group	Total Number
		FiO_2's	Duration	Type		
Cheney (1980)	Canine	0.21 and 0.50	4d	IC Oleic Acid	7 and 6 per FiO_2 respectively	13
		0.21 and 0.50	8d	IC Oleic Acid	5 and 6 per FiO_2 respectively	11
Rinaldo (1985)	Rats	0.21 and 0.60	1, 3, or 6d	IP LD LPS	6-8 per FiO_2 and duration (BAL cells)	36-48
		0.21 and 0.60	1d	IP LD or HD LPS	6-8 per FiO_2 and LPS dose (BAL cells and survival)	36-48
Garner (1988)	Rats	0.21, 0.40, and 0.80	7d	CLP	39 per FiO_2	117
		0.21, 0.40, and 0.80	7d	Sham	6-7 per FiO_2	18-21
Cantor (1990)	Hamster	0.21 and 0.60	4d	Elastase	3 per FiO_2	6
		0.21 and 0.60	4d	NS	3 per FiO_2	6
		0.21 and 0.60	7d	Elastase	8 per FiO_2	16
		0.21 and 0.60	7d	NS	3 and 4 per FiO_2 respectively	7
		0.21 and 0.60	35d	Elastase	9 per FiO_2	18
		0.21 and 0.60	35d	NS	4 per FiO_2	8
Knight (2000)	Rabbits	0.21 and 0.50	1d	No HCL	5 per FiO_2	10
		0.21 and 0.50	1d	IT HCL	5 per FiO_2	10
Nara (2004)	Mice	0.21 and 0.90	2d	IT *L. pneumo.* $5x10^7$, $1.6x10^7$, $5x10^6$, or $1.6x10^6$	8-12 per FiO_2 and *L. pneumo.* dose (survival study)	64-96
		0.21 and 0.50	2 or 4d	IT *L. pneumo.* $5x10^6$	8-12 per FiO_2 and duration (survival study)	32-48
		0.21 and 0.70	2 or 4d	IT *L. pneumo.* $5x10^6$	8-12 per FiO_2 and duration (survival study)	32-48
		0.21 and 0.90	2 or 4d	IT *L. pneumo.* $5x10^6$	8-12 per FiO_2 and duration (survival study)	32-48
		0.21	4d	None	8-12 (survival study)	8-12
		0.21, 0.50, and 0.90	2d	IT *L. pneumo.* $1.6x10^6$	5 per FiO_2 (non-survival studies)	15
		0.21, 0.50 and 0.90	2d	None	5 per FiO_2 (non-survival studies)	15
Sun (2006)	Rats	0.21, 0.40 and 1.0	1d	IT *K. pneumo.*	9, 8, and 9 respectively	26
		0.21	1d	NS	8	8
Aggarwal (2010)	Mice	0.21 and 0.27	4d	IT LPS	3-4 per FiO_2	6-8
		0.27	4d	IT Water	3-4	3-4
		0.21 and 0.60	0.5, 2, 3, or 4d	IT LPS	3-4 or 3-5 per FiO_2 per duration per parameter	UC
		0.21 and 0.60	0.5, 2, 3, or 4d	IT Water	3-4 or 3-5 per FiO_2 per duration per parameter	UC
Rodriguez-Gonzalez (2014)	Rats	0.21, 0.40, 0.60, and 1.0	1d	CLP	8 per FiO_2	32
		0.21, 0.40, 0.60, and 1.0	1d	Sham	4 per FiO_2 (survival study only)	16
Garcia-Laorden (2020)	Rats	0.21, 0.40, 0.60, and 1.0	1d	CLP	12, 8, 9 and 10 per FiO_2 respectively	39
		0.21, 0.40, 0.60, and 1.0	1d	Sham	4 per FiO_2	16
		0.21, 0.40, 0.60, and 1.0	1d	Healthy	6 per FiO_2	24

CLP – cecal ligation and puncture; HD – high dose; HCL – hydrochloric acid; IC – intracardiac; IT – intratracheal; *K. pneumo.* – *Klebsiella pheumophilia*; *L. pneumo* – *Legionella pneumophilia;* LD – low dose; LPS – lipopolysaccharide; NS – normal saline; Sham – sham CLP; UC – unclear

Table S2. Results of lung injury measures reported in O_2 + nonO_2 inflammatory challenge studies for groups exposed to FiO_2s≤0.60 and >0.21 or FiO_2=0.21

Reference	Species	Increased $Fi0_2$ Studied		Inflammatory challenge		Measures*		Reported Results for Groups	
		Level	Duration	Type	Time	Type	Variance	FiO_2=0.21	FiO_2≤0.60 and >0.21
**Lung pathology scores**									
Cantor (1990)	Rats	0.60	35d	IT Elastase	0h	Airspace size (um)†	SE	75±5	125±10‡
Sun (2006)	Rats	0.40	1d	IT *K. Pneumo.*	0h	Lung Injury Score†	SD	4.7±0.9	3.2±1.2‡
Aggarwal (2010)	Mice	0.60	2d	IT LPS	0h	Histology score†	SE	1.8±0.1	1.8±0.2
Aggarwal (2010)	Mice	0.60	3d	IT LPS	0h	Histology score†	SE	1.0±0.3	3.5±0.5‡
Aggarwal (2010)	Mice	0.60	4d	IT LPS	0h	Histology score†	SE	1.0±0.3	5.5±2.0‡
Garcia-Laorden (2020)	Rats	0.40	1d	CLP	-18h	Total lung pathology score†	IQR	5 (2, 7)	3 (2, 4)
Garcia-Laorden (2020)	Rats	0.60	1d	CLP	-18h	Total lung pathology score†	IQR	5 (2, 7)	4(3, 5)
**Lung weights**									
Cheney (1980)	Canine	0.50	4d¶	OA	-4h	Gm H_20/gm dry wgt	SD	4.66±0.61	4.91±0.66
Cheney (1980)	Canine	0.50	8d¶	OA	-4h	Gm H_20/gm dry wgt	SD	4.12±0.27	4.10±049
Knight (2000)	Rabbits	0.50	1d	IT HCL	0h	Gm/kg body wgt	SE	7.5±1.5	13±2.5‡
Nara (2004)	Mice	0.50	4d	IT *L. pneumo.*	0h	Lung wgt (g)†	UC	0.19±0.01	0.24±0.01‡
Sun (2006)	Rat	0.40	1d	IT *K. pneumo.*	0h	W/D	SD	4.2±04	4.3±0.2
Aggarwal (2010)	Mice	0.60	4d	IT LPS	0h	Lung wgt/baseline wgt†	SE	2.3±0.1	2.6±0.1‡
**Arterial oxygenation**									
Cheney (1980)	Canine	0.50	4d¶	IT *Oleic* acid	0h	PaO_2 (mmHg)†	SD	65±2	60±2
Cheney (1980)	Canine	0.50	8d¶	IT Oleic acid	0h	PaO_2 (mmHg)††	SD	75±2	70±3
Knight (2000)	Rabbits	0.50	1d	IT HCL	0h	P:F†	SE	260±30	100±50‡
Sun (2006)	Rats	0.40	1d	IT *K. pneumo.*	0h	PaO_2 (mmHg) †	SD	56.4±16.7	82.6±20.5
Aggarwal (2010)	Mice	0.60	4d	IT LPS	0h	PaO_2 (mmHg)††	SE	100±1	80±1‡
**Lung lavage protein**									
Sun (2006)	Rats	0.40	1d	IT *K. pneumo.*	0h	BAL Protein (mg/kg)	SD	32.8±22.1	30.5±16.3
Aggarwal (2010)	Mice	0.27	4d	IT LPS	0h	BAL Protein (ug/mL) †	SE	240±20	240±60
Aggarwal (2010)	Mice	0.60	2d	IT LPS	0h	BAL Protein (ug/mL) †	SE	400±20	500±20
Aggarwal (2010)	Mice	0.60	3d	IT LPS	0h	BAL Protein (ug/mL) †	SE	400±20	520±50‡
Aggarwal (2010)	Mice	0.60	4d	IT LPS	0h	BAL Protein (ug/mL) †	SE	280±10	400±40‡
Garcia- Laorden (2020)	Rats	0.40	1d	CLP	-18h	BAL Protein (mg/dL) †	IQR	10 (5,20)	10 (3,22)
Garcia- Laorden (2020)	Rats	0.60	1d	CLP	-18h	BAL protein (mg/dL) †	IQR	10 (5,20)	15 (12, 29)
**Bronchoalveolar lavage surface tension**									
Knight (2000)	Rabbits	0.50	1d	IT HCL	0h	BAL surface tension (mN/m) †	SE	4±2	28±2§
Sun (2006)	Rats	0.40	1d	IT *K. pneumo.*	0h	BAL surface tension (mN/m)	SD	35.2±3.4	36.0±2.4

**Bacteria counts**									
Sun (2006)	Rat	0.40	1d	IT *K. pneumo.*	0h	BAL logCFU/rat†	SD	4.2±0.2	5±0.1
Sun (2006)	Rat	0.40	1d	IT *K. pneumo.*	0h	Lung tissue logCFU/rat†	SD	7±0.1	7.2±01
Rodriguez-Gonzalez (2014)	Rat	0.40	1d	CLP	0h	Number of infected samples†, ††	SD	2.4±0.3	2.2±0.4
Rodriguez-Gonzalez (2014)	Rat	0.60	1d	CLP	0h	Number of infected samples†, ††	SD	2.4±0.3	3.1±03‡
**Other potential measures of lung injury or dysfunction**									
Cheney (1980)	Canine	0.50	4d¶	IT *Oleic* acid	0h	QVa/QT(%)†	SD	21±5	9±4
Cheney (1980)	Canine	0.50	8d¶	IT *Oleic* acid	0h	QVa/QT(%)†	SD	20±10	10±6
Cheney (1980)	Canine	0.50	4d¶	IT *Oleic* acid	0h	$PaCO_2$ (mmHg)	SD	32±4	36±7‡
Cheney (1980)	Canine	0.50	8d¶	IT *Oleic* acid	0h	$PaCO_2$ (mmHg)	SD	31±2	36±5‡
Cheney (1980)	Canine	0.50	4d¶	IT *Oleic* acid	0h	PAP	SD	15±4	13±3
Cheney (1980)	Canine	0.50	8d¶	IT *Oleic* acid	0h	PAP	SD	15±2	14±2
Cantor (1990)	Rats	0.60	7d	IT Elastase	0h	C^{14} lysine elastin cross link uptake†, #	SE	550±50	850±100‡
Knight (2000)	Rabbits	0.50	1d	IT HCL	0h	Type-2 cell H^3choline uptake †, **	SE	0.46±0.5	0.25±0.6§
Knight (2000)	Rabbits	0.50	1d	IT HCL	0h	TLV/body wgt (mL/kg) †	SE	35±5	10±5§
Knight (2000)	Rabbits	0.50	1d	IT HCL	0h	$dV/dP_{(25-75\%)}$† (ml/kg/cmH_2O)	SE	1.4±1	0.6±0.2§
Sun (2006)	Rats	0.40	1d	IT *K. pneumo.*	0h	BAL DSPC/TPL	SD	55.1±4.3	47.8±2.2§

*Measure descriptions and units are those provided in studies; † Means or medians and variances or interquartile ranges determined from figures; ‡ $p \leq 0.05$ as reported in study; § $p \leq 0.01$ as reported in study; ¶ data reported on days 4 and 8 when an initial animal group and then remaining animals respectively were sacrificed; # counts per min/gm dry lung; ** (nmol/10^6cells/h); ††Number of infected samples averaged across 8 samples each from blood, BAL, peritoneal fluid, urine and meninges

BAL – bronchoalveolar lavage; CLP – cecal ligation and puncture; DSPC/TPL – desaturated phosphatidyl choline to total phospholipid ratio; dV/dP – change in volume over change in pressure; HCL – hydrochloric acid; IP – intraperitoneal; IQR – interquartile range; IT – intratracheal; LIS – lung injury, tissue pathology or histology score; LPS – lipopolysaccharide; MPO – myeloperoxidase; NR- Not Reported; PAP – pulmonary artery pressure; P:F – PaO_2 to FiO_2 ratio; PMN – polymorphonuclear cells; QVa/QT – venous admixture; ROS – reactive oxygen species; SD – Standard Deviation; SE – Standard Error; TLV – total lung volume; UC – unclear; W/D – wet to dry weight ratio; wgt – weight

Table S3. Results of lung injury and immune response measures reported in O_2 + nonO_2 inflammatory challenge studies for groups exposed to FiO_2 >0.60 or FiO_2=0.21

Author (y)	Species	Increased FiO2 Studied		Inflammatory Challenge		Measures*		Reported Results for Groups	
		Level	Duration	Type	Time	Type	Variance	FiO_2=0.21	FiO_2>0.60
Lung Injury									
**Lung pathology scores**									
Garcia-Laorden ('20)	Rats	1.0	1d	CLP	-18h	Total lung pathology score †	IQR	5 (2, 7)	5 (3, 7)
**Lung weights**									
Nara (2004)	Mice	0.90	2d	IT *L. pneumo*	0h	Lung wgt (g) †	NR	019±0.01	0.28±0.02‡
Sun (2006)	Rat	0.98	1d	IT *K. pneumo*	0h	W/D	SD	4.2±04	5.1±0.3
**Arterial oxygenation**									
Sun (2006)	Rats	0.90	1d	IT *K. pneumo.*	0h	PaO2	SD	56.4±16.7	76.2±19.3
**Lung lavage protein**									
Sun (2006)	Rats	0.98	1d	IT *K. pneumo.*	0h	BAL Protein (mg/kg)	SD	32.8±22.1	37.6±19.3
Garcia- Laorden (2020)	Rats	1.0	1d	CLP	-18h	BAL Protein (mg/dL) †	IQR	10 (5,20)	18 (15, 27)
**BAL surface tension**									
Sun (2006)	Rats	0.98	1d	IT *K. pneumo.*	0h	BAL surface tension (mN/m)	SD	35.2±3.4	37.0±5.6
**Bacteria counts**									
Sun (2006)	Rat	0.98	1d	IT *K. pneumo.*	0h	BAL logCFU/rat †	SD	4.2±0.2	5±0.1
Sun (2006)	Rat	0.98	1d	IT *K. pneumo.*	0h	Lung tissue logCFU/rat†	SD	7±0.1	7.2±01
Rodriguez-Gonzalez ('14)	Rat	1.0	1d	CLP	0h	Number of infected samples†,††	SD	2.4±0.3	4.4±0.1‡
**Other potential measures of lung injury or dysfunction**									
Sun (2006)	Rats	0.98	1d	IT *K. pneumo.*	0h	BAL DSPC/TPL	SD	55.1±4.3	42.0±4.3§
Immune Response									
**BAL or lung PMNs**									
Sun (2006)	Rats	0.90	1d	IT *K. pneumo.*	0h	BAL PMNs (cells/mLx106)	SD	1.99±0.96	2.16±0.68
Garcia- Laorden (2020)	Rats	1.0	1d	CLP	-18h	BAL% PMNs	IQR	6 (5,7)	2(1,3)
**BAL or lung tissue MACs**									
Sun (2006)	Rats	0.90	1d	IT *K. pneumo.*	0h	BAL MACs (x106/ml)	SD	0.29±0.28	0.36±013
**BAL, lung Tissue or serum cytokines**									
Sun (2006)	Rats	0.90	1d	IT *K. pneumo.*	0h	Lung tissue TNFα†	SD	250±75	200±50
Rodriguez-Gonzalez (2014)	Rat	1.0	1d	CLP	0h	Serum BAL IL-6 (pg/mL) †	SD	390±20	720±10‡, ¶
Rodriguez-Gonzalez (2014)	Rat	1.0	1d	CLP	0h	Serum BAL TNFα (pg/mL) †	SD	75±20	60±5

Rodriguez-Gonzalez (2014)	Rat	1.0	1d	CLP	0h	Serum BAL IL-10 (pg/mL) †	SD	1000±200	3500±2000‡, ¶
Garcia- Laorden (2020)	Rats	1.0	1d	CLP	-18h	Serum IL-6 (pg/mL)	IQR	400 (329, 454)	724 (650, 744)
**Changes in other immune response parameters**									
Sun (2006)	Rats	0.90	1d	IT *K. pneumo.*	0h	Lung MPO (U/g) †	SD	6±1	3.5=0.5
Sun (2006)	Rats	0.90	1d	IT *K. pneumo.*	0h	Lung ICAM-1 (pg/ml) †	SD	190±80	180±70
Sun (2006)	Rats	0.90	1d	IT *K. pneumo.*	0h	Lung NF-κβ (du) †	SD	40±2	40±3
Sun (2006)	Rats	0.90	1d	IT *K. pneumo.*	0h	Lung iNOS (U/mg/protein) †	SD	0.55±0.2	0.35±0.1
Sun (2006)	Rats	0.90	1d	IT *K. pneumo.*	0h	BAL NOx- (umol/L)	SD	63±17	83±23
Rodriguez-Gonzalez (2014)	Rat	1.0	1d	CLP	0h	Serum ROS†	SD	10±2	26±6‡
Garcia- Laorden (2020)	Rats	1.0	1d	CLP	-18h	S100B (ug/L) †	IQR	0.04(0.01, 0.01)	0.09(0.01,0.05)§

*Measure description and units are those provided in studies; † Means or medians and variances or interquartile ranges determined from figures; ‡ p≤0.05 as reported in study; § p≤0.01 as reported in study; ¶data reported on days 4 and 8 when an initial animal group and then remaining animals respectively were sacrificed; # counts per min/gm dry lung; ** (nmol/106cells/h); ††Number of infected samples averaged across 8 samples each from blood, BAL, peritoneal fluid, urine and meninges

BAL – bronchoalveolar lavage; CLP – cecal ligation and puncture; DSPC/TPL – desaturated phosphatidyl choline to total phospholipid ratio; HCL – hydrochloric acid; ICAM – ICAM – intracellular adhesion molecule; iNOS – inducible nitric oxide synthase; IL – interleukin; IP – intraperitoneal; IQR – interquartile range; IT – intratracheal; LIS – lung injury; tissue pathology or histology score; LPS – lipopolysaccharide; MAC – macrophage; MPO – myeloperoxidase; NO – nitric oxide; NR- Not Reported; P:F – PaO_2 to FiO_2 ratio; PMN – polymorphonuclear cells; ROS – reactive oxygen species; SD- Standard Deviation; SE- Standard Error; TNF – tumor necrosis factor; UC – unclear; W/D – wet to dry weight ratio

Table S4. Results of immune response measures reported in O_2 + nonO_2 inflammatory challenge studies for groups exposed to FiO_2s≤0.60 and >0.21

Author (y)	Species	Increased FiO_2 Studied		Inflammatory Challenge		Measures*		Reported Results for Groups	
		Level	Duration	Type	Time	Type	Variance	FiO_2=0.21	FiO_2≤0.60 and >0.21
**BAL or Lung PMNs**									
Rinaldo (1985)	Rats	0.60	1d	LD IP LPS	0h	PMNs/lung (x10-5) †	SEM	8±1	4.5±1.5‡
Rinaldo (1985)	Rats	0.60	3d	LD IP LPS	0h	PMNs/lung (x10-5) †	SEM	3.5±1	1.5±1
Rinaldo (1985)	Rats	0.60	6d	LD IP LPS	0h	PMNs/lung (x10-5) †	SEM	1.5±1	0.5±0.5
Rinaldo (1985)	Rats	0.60	1d	LD IP LPS	0h	BAL %PMNs †	SEM	17±2	9±3
Rinaldo (1985)	Rats	0.60	1d	HD IP LPS	0h	BAL %PMNs †	SEM	34±4	12±2§
Cantor (1990)	Rats	0.60	2d	IT Elastase	0h	BAL %PMNs	SEM	2.9±1.1	27.7±6.0‡
Sun (2006)	Rats	0.40	1d	IT *K. pneumo.*	0h	BAL PMNs (cells/mLx106)	SD	1.99±0.96	2.22±1.55
Aggarwal (2010)	Mice	0.27	4d	IT LPS	0h	BAL PMNs (cells/mLx106) †	SE	0.55±0.1	0.4±0.1
Aggarwal (2010)	Mice	0.60	2d	IT LPS	0h	BAL PMNs (cells/mLx106) †	SE	1.5±0.1	1.7±0.2
Aggarwal (2010)	Mice	0.60	3d	IT LPS	0h	BAL PMNs (cells/mLx106) †	SE	0.9±0.1	1.7±0.2‡
Aggarwal (2010)	Mice	0.60	4d	IT LPS	0h	BAL PMNs (cells/mLx106) †	SE	0.5±0.1	1.0±0.2‡
Garcia- Laorden (2020)	Rats	0.40	1d	CLP	-18h	BAL %PMNs	IQR	6 (5,7)	12 (7, 13)
Garcia- Laorden (2020)	Rats	0.60	1d	CLP	-18h	BAL %PMNs	IQR	6 (5,7)	3(2, 3)
**BAL or lung tissue MACs**									
Rinaldo (1985)	Rats	0.60	1d	LD IP LPS	0h	MACs/lung (x10-6) †	SEM	4.0±0.2	4.5±05
Rinaldo (1985)	Rats	0.60	3d	LD IP LPS	0h	MACs/lung (x10-6) †	SEM	4.5±0.5	4.5±0.3
Rinaldo (1985)	Rats	0.60	6d	LD IP LPS	0h	MACs/lung (x10-6) †	SEM	4.8±0.5	5.1±05
Sun (2006)	Rats	0.40	1d	IT *K. pneumo.*	0h	BAL MACs (x106/ml)	SD	0.29±0.28	0.39±0.23
Aggarwal (2010)	Mice	0.60	2d	IT LPS	0h	BAL MACs (cells/mLx106) †	SE	0.10±0.02	0.08±0.02
Aggarwal (2010)	Mice	0.60	3d	IT LPS	0h	BAL MACs (cells/mLx106) †	SE	0.26±0.02	0.16±0.02‡
Aggarwal (2010)	Mice	0.60	4d	IT LPS	0h	BAL MACs (cells/mLx106) †	SE	0.40±0.08	0.18±0.01‡
**BAL, Lung Tissue or Serum Cytokines**									
Sun (2006)	Rats	0.40	1d	IT *K. pneumo.*	0h	Lung tissueTNFα†	SD	250±75	150±25
Aggarwal (2010)	Mice	0.27	4d	IT LPS	0h	BAL KC (pg/mL) †	SE	75±15	70±17
Aggarwal (2010)	Mice	0.27	4d	IT LPS	0h	BAL MIP-2 (pg/ml) †	SE	10±5	10±1
Aggarwal (2010)	Mice	0.27	4d	IT LPS	0h	BAL LIX (pg/mL) †	SE	2300±200	2700±100
Aggarwal (2010)	Mice	0.60	2d	IT LPS	0h	BAL TNFα (pg/ml) †	SE	90±10	150±5‡

Aggarwal (2010)	Mice	0.60	3d	IT LPS	0h	BAL TNFα (pg/ml) †	SE	30±5	50±5‡
Aggarwal (2010)	Mice	0.60	4d	IT LPS	0h	BAL TNFα (pg/ml) †	SE	10±5	45±5‡
Aggarwal (2010)	Mice	0.60	4d	IT LPS	0h	BAL TGFβ (pg/mL) †	SE	210±25	125±2‡
Aggarwal (2010)	Mice	0.60	2d	IT LPS	0h	BAL IL-10 (pg/mL) †	SE	20±1	30±6
Aggarwal (2010)	Mice	0.60	4d	IT LPS	0h	BAL IL-10 (pg/mL) †	SE	8±1	12±1
Aggarwal (2010)	Mice	0.60	1d	IT LPS	0h	BAL KC (pg/mL) †	SE	160±20	500±90‡
Aggarwal (2010)	Mice	0.60	2d	IT LPS	0h	BAL KC (pg/mL) †	SE	250±50	400±10‡
Aggarwal (2010)	Mice	0.60	3d	IT LPS	0h	BAL KC (pg/mL) †	SE	150±10	160±5
Aggarwal (2010)	Mice	0.60	4d	IT LPS	0h	BAL KC (pg/mL) †	SE	100±50	120±10
Aggarwal (2010)	Mice	0.60	1d	IT LPS	0h	BAL MIP-2 (pg/ml) †	SE	165±50	150±50
Aggarwal (2010)	Mice	0.60	2d	IT LPS	0h	BAL MIP-2 (pg/ml) †	SE	140±10	220±20‡
Aggarwal (2010)	Mice	0.60	3d	IT LPS	0h	BAL MIP-2 (pg/ml) †	SE	50±10	110±10‡
Aggarwal (2010)	Mice	0.60	4d	IT LPS	0h	BAL MIP-2 (pg/ml) †	SE	10±1	40±2
Aggarwal (2010)	Mice	0.60	1d	IT LPS	0h	BAL LIX (pg/mL) †	SE	1800±1000	3000±500
Aggarwal (2010)	Mice	0.60	2d	IT LPS	0h	BAL LIX (pg/mL) †	SE	2200±500	4200±1000
Aggarwal (2010)	Mice	0.60	3d	IT LPS	0h	BAL LIX (pg/mL) †	SE	1800±1000	5000±800‡
Aggarwal (2010)	Mice	0.60	4d	IT LPS	0h	BAL LIX (pg/mL) †	SE	2100±200	3200±200
Rodriguez-Gonzalez (2014)	Rat	0.40	1d	CLP	0h	Serum IL-6 (pg/mL) †	SD	390±20	400±20
Rodriguez-Gonzalez (2014)	Rat	0.60	1d	CLP	0h	Serum IL-6 (pg/mL) †	SD	390±20	540±20‡
Rodriguez-Gonzalez (2014)	Rat	0.40	1d	CLP	0h	Serum TNFα (pg/mL) †	SD	75±20	35±5‡
Rodriguez-Gonzalez (2014)	Rat	0.60	1d	CLP	0h	Serum TNFα (pg/mL) †	SD	75±20	35±10‡
Rodriguez-Gonzalez (2014)	Rat	0.40	1d	CLP	0h	Serum IL-10 (pg/mL) †	SD	1000±200	700±200
Rodriguez-Gonzalez (2014)	Rat	0.60	1d	CLP	0h	SerumIL-10 (pg/mL) †	SD	1000±200	700±200
Garcia- Laorden (2020)	Rats	0.40	1d	CLP	-18h	Serum IL-6 (pg/mL)	IQR	400 (329, 454)	426 (414, 472)
Garcia- Laorden (2020)	Rats	0.60	1d	CLP	-18h	Serum IL-6 (pg/mL)	IQR	400 (329, 454)	555 (517, 571)
**Changes in other Immune Response Parameters**									
Cantor (1990)	Rats	0.60	2d	IT Elastase	0h	Total BAL leukocytes (x106)	SEM	4.8±1.0	3.9±0.3
Sun (2006)	Rats	0.40	1d	IT *K. pneumo.*	0h	Lung MPO (U/g) †	SD	6±1	5.5±0.5
Sun (2006)	Rats	0.40	1d	IT *K. pneumo.*	0h	Lung ICAM-1 (pg/ml) †	SD	190±80	240±50
Sun (2006)	Rats	0.40	1d	IT *K. pneumo.*	0h	Lung NF-κβ (du) †	SD	40±2	43±2
Sun (2006)	Rats	0.40	1d	IT *K. pneumo.*	0h	Lung iNOS (U/mg/protein) †	SD	0.55±0.2	0.68±0.2
Sun (2006)	Rats	0.40	1d	IT *K. pneumo.*	0h	BAL NOx- (umol/L) †	SD	63±17	65±21
Aggarwal (2010)	Mice	0.60	1d	IT LPS	0h	BAL Lyms (cells/mLx104) †	SE	0.10±0.01	0.10±0.01
Aggarwal (2010)	Mice	0.60	2d	IT LPS	0h	BAL Lyms (cells/mLx104) †	SE	0.3±0.1	0.3±0.2
Aggarwal (2010)	Mice	0.60	3d	IT LPS	0h	BAL Lyms (cells/mLx104) †	SE	1.4±04	0.4±0.1‡

Aggarwal (2010)	Mice	0.60	4d	IT LPS	0h	BAL Tregs (number) †	SE	450±25	260±50‡
Aggarwal (2010)	Mice	0.60	2d	IT LPS	0h	PMN apop(%Annexin) †	SE	9±2	13±7
Aggarwal (2010)	Mice	0.60	3d	IT LPS	0h	PMN apop(%Annexin) †	SE	15±4	19±3
Aggarwal (2010)	Mice	0.60	4d	IT LPS	0h	PMN apop(%Annexin) †	SE	22±3	21±2
Rodriguez-Gonzalez (2014)	Rat	0.40	1d	CLP	0h	Serum ROS†	SD	10±2	9±1
Rodriguez-Gonzalez (2014)	Rat	0.60	1d	CLP	0h	Serum ROS†	SD	10±2	14±4
Garcia- Laorden (2020)	Rats	0.40	1d	CLP	-18h	S100B (ug/L) †	IQR	0.04 (0.01, 0.01)	0.03(0.01,0.02)
Garcia- Laorden (2020)	Rats	0.60	1d	CLP	-18h	S100B (ug/L) †	IQR	0.04 (0.01, 0.01)	0.05(0.02,0.02)

*Measure descriptions and units are those provided in studies; † Means or medians and variances or interquartile ranges determined from figures; ‡ p≤0.05 as reported in the study; § p≤0.01 as reported in the study

apop – apoptosis; BAL – bronchoalveolar lavage; CLP – cecal ligation and puncture; du – densitometric units; HCL – hydrochloric acid; HD – high dose; IP – intraperitoneal; ICAM-1 – intracellular adhesion molecule-1; IL – interleukin; IT – intratracheal; LD – low dose; KC – keratinocyte derived chemokine; *K. pneumo* – *Klebsiella pneumonia*; LIS – lung injury, tissue pathology or histology score; LIX – CXCL5; LPS – lipopolysaccharide; Lyms – lymphocytes; MAC – macrophage; MIP – macrophage inflammatory protein; MPO – myeloperoxidase; NR- not reported; P:F – PaO2 to FiO2 ratio; PMN – polymorphonuclear cells; ROS – reactive oxygen species; SD – Standard Deviation; SE – Standard Error; TGF – transforming growth factor; TNF – tumor necrosis factor; TREG – T-regulatory cell; %Annexin – %Annexin V+/7-AAD-

Table S5. Animal numbers for O_2 only studies

Author (y)	Type of Animal Studied	Oxygen Regimens Compared		Animal Number per Group	Total Number
		FiO_2s	Duration		
Hayatdavoudi (1981)	Rats	0.21, 0.60, and 0.85	3d	40 per FiO_2 (O_2 tolerance to FiO2=1.0)	120
		0.21, 0.60, and 0.85	7d	24, 8, and 16 per FiO_2 respectively (biochemical changes)	48
		0.21 and 0.60	7d	4 per FiO_2 (Pulmonary SOD study)	8
		0.21 and 0.60	7d	44 per FiO_2 (metabolism study	88
		0.21 and 0.60	7d	8 and 6 respectively (histology study)	14
		0.21 and 0.60	7d	8 and 6 respectively (BAL study)	14
		0.21 and 0.60	7d	16 and 14 respectively (P/V study)	30
		0.21 and 0.60	7d	35 per FiO_2 (Mechanical Ventilation study)	70
Coursin (1987)	Rats	0.21, 0.50 0.65, and 0.8*	4,7,11,14,21, 28, or 42d	28 per FiO_2	112
		0.21, 0.50 0.65, and 0.8	6w	6,4,3 and 5 respectively (O_2 tolerance to FiO2=1.0)	18
Van Klaveren(1997)	Rats	0.21, 0.60, and 0.85	7d	5-7 per FiO_2 (Pulmonary index study)	15-21
		0.21, 0.60, and 0.85	7d	5-7 per FiO_2 (Lung homogenate study)	15-21
		0.21, 0.60, and 0.85	7d	5-7 per FiO_2 (BAL study)	15-21
		0.21, 0.60, and 0.85	7d	5-7 per FiO_2 (Antioxidant study)	15-21
Belik (2003)	Mice	0.21 and 0.60	14d	27 and 43 respectively (force generation study)	70
		0.21 and 0.60	14d	20 and 30 respectively (relaxation study)	50
Nelin (2003)	Rats	0.21, 0.50, and 0.90	57h	6,10, and 11 respectively (survival study)	32-48
Gan (2011)	Rats	0.21, 0.60, and 0.85	7d	38, 38, and 41 respectively (body/lung weight study)	117
		0.21, 0.60, and 0.85	7d	4, 5, and 5 respectively (DQ infusion study)	14
		0.21, 0.60, and 0.85	7d	5 per FiO_2 (DQ + AA infusion study)	15
		0.21, 0.60, and 0.85	7d	6, 6, 4 respectively (DQH_2 + DIC study)	14
		0.21, 0.60, and 0.85	7d	4 per FiO_2 (DQH_2 + DIC + ROT study)	12
		0.21, 0.60, and 0.85	7d	7, 10, and 7 respectively (NQO1 study)	24
		0.21, 0.60, and 0.85	7d	5, 5, and 7 respectively (Mitochondrial study)	17
		0.21, 0.60, and 0.85	7d	8, 5, and 7 respectively (GSH + GSSG study)	20
Audi (2012)	Rats	0.21, 0.60, and 0.85	7d	13, 8, and 12 respectively (lung weight study)	33
		0.21, 0.60, and 0.95	2 or 7d	19, 7, and 7 respectively (HMPAO study)	33
		0.21, 0.60, and 0.95	2 or 7d	13, 8, and 7 respectively (antioxidant study)	28
		0.21, 0.60, and 0.95	2 or 7d	4, 5, and 5 respectively (mitochondrial study)	14

Hackney (1975)	Monkey (Squirrel)	0.21, 0.60, and 0.80	2, 4, or 8d	12, 16, 16 respectively	44
Holm (1987)	Rabbits (NZW)	0.21 and 0.60	21d	7 per FiO_2 (lung mechanics study)	14
		0.21 and 0.60	21d	6 per FiO_2 (BAL study)	12
		0.21 and 0.60	21d	6 per FiO_2 (alveolar permeability study)	12
		0.21 and 0.60	21d	5 and 8 respectively (laser flow study)	13
Nickerson (1990)	Rabbits (NZW)	0.21 and 0.60	21 d	4 per FiO_2	8
Rister (1983)	Guinea Pigs	0.21, 0.30, 0.40, 0.50, 0.60, 0.70, 0.80	90h	UC	UC
Hesse (2004)	Mice (C57BL/6J)	0.21, 0.60, and 0.95	3d	7 per FiO_2	UC
Lagishetty (2014)	Mice (C57BL/6J)	0.21, 0.50, 0.75 and 1.0	3d	6, 6 ,6, and 18 respectively	36
Nylen (1993)	Hamsters (Syrian Gold)	0.21 and 0.60	7d, 21d, 3m	10 per group per time period	60

*Results inferred from figures which only gives n for control group

AA – antimycin A; BAL – bronchioalveolar lavage; DQ – duroquinone; DQH_2 – durohydroquinone; DIC – dicumarol; GSH-glutathione; GSSG-oxidized glutathione; HMPAO – technetium-labeled-hexamethylpropyleneamine oxim (trapped in the lung by GSH); NQO1 – NAD(P)H:quinone oxidoreductase 1; NZW – New Zealand White; P/V – pressure volume study; ROT – rotenone; SOD – Superoxide dismutase; UC – unclear

Table S6. Body weights following oxygen exposure for O_2 only studies								
Author (y)	Animals Type	Increased FiO_2 Studied		FiO_2=0.21 Group		Increased FiO_2 Group		Variance Type
		Level	Duration	Number	Wgt (g)	Number	Wgt (g)	
**FiO_2≤0.60 and >0.21**								
Hayatdavoudi (1981)	Rat	0.60	7d	8	395±3	8	380±4	SE
Coursin (1987)*	Rat	0.50	7d	4	230±10	4	220±10	SE
Coursin (1987)*	Rat	0.50	14d	4	275±5	4	263±10	SD
Coursin (1987)*	Rat	0.50	21d	4	313±15	4	310±10	SD
Coursin (1987)*	Rat	0.50	28d	4	353±12	4	365±15	SD
Coursin (1987)*	Rat	0.50	42d	4	390±15	4	425±23	SD
Nylen (1993)	Hamster	0.60	7d	10	111±8	10	110±11	SD
Nylen (1993)	Hamster	0.60	21d	9	128±11	9	109±9	SD
Nylen (1993)	Hamster	0.60	90d	10	162±10	6	88±19	SD
Van Klaveren (1997)	Rats	0.60	7d	6	212±10	6	208±7	SD
Nelin (2003)	Rats	0.50	2.5d	6	298±3	10	300±3	SE
Hesse (2004)	Mice	0.60	3d	7	28.1±0.9	7	28.1±0.3	SE
Audi (2011)	Rats	0.60	7d	5	345.8±9.9	33	355.1±2.7	SE
**FiO_2>0.60**								
Hayatdavoudi (1981)	Rat	0.85	7d	16	397±3	16	323±5	SE
Coursin (1987)*	Rat	0.65	7d	4	230±10	4	220±10	SD
Coursin (1987)*	Rat	0.65	14d	4	275±5	4	265±5	SD
Coursin (1987)*	Rat	0.65	21d	4	313±15	4	305±10	SD
Coursin (1987)*	Rat	0.65	28d	4	353±12	4	335±15	SD
Coursin (1987)*	Rat	0.65	42d	4	390±15	4	387±13	SD
Coursin (1987)*	Rat	0.80	7d	4	230±10	4	185±10	SD
Coursin (1987)*	Rat	0.80	14d	4	275±5	4	203±10	SD
Coursin (1987)*	Rat	0.80	21d	4	313±15	4	212±12	SD
Coursin (1987)*	Rat	0.80	28d	4	353±12	4	243±13	SD
Coursin (1987)*	Rat	0.80	42d	4	390±115	4	266±10	SD
Van Klaveren (1997)	Rats	0.85	7d	6	212±10	6	163±9	SD
Nelin (2003)	Rats	0.90	2.5d	6	298±3	11	288±4	SE
Hesse (2004)	Mice	>0.95	3d	7	28.1±0.9	7	23.2±0.6	SE
Audi (2011)	Rats	0.85	7d	5	345.8±9.9	33	268.1±3.2	SE

*Results determined from figures

SD – standard deviation; SE – standard error; Wgt – weight

Table S7. Results of lung injury measures reported in O_2 only studies for groups exposed to FiO_2s≤0.60 and >0.21 or FiO_2=0.21

Author (y)	Species	Increased FiO2 Studied		Measures*		Reported Results for Groups	
		Level	Duration	Type	Variance	FiO2=0.21	FiO2 ≤0.60 and >0.21
**Lung weights**							
Hayatdavoudi, (1981)	Rat	0.60	7d	LW(g)	SE	1.56±0.03	1.53±0.05
Holm, (1987)	Rabbit	0.60	21d	W/D	SE	4.10±0.30	5.60±0.30‡
Nylen, (1993)	Hamster	0.60	7d	LW(g)	SD	0.38±0.05	0.39±0.04
Nylen, (1993)	Hamster	0.60	21d	LW(g)	SD	0.39±0.03	0.43±0.08
Nylen, (1993)	Hamster	0.60	90d	LW(g)	SD	0.49±0.08	0.35±0.07§
Van Klaveren, (1997)	Rat	0.60	7d	W/D (gm H20/100 gm dry wgt)	SD	0.91±0.07	1.00±0.13
Nelin, (2003)	Rat	0.50	2.4d	LW(g)	SE	1.23± 0.01	1.48±0.06
Gan (2011)	Rat	0.60	7d	W/D	SE	5.56±0.09	5.45±0.05
Audi, (2012)	Rat	0.60	7d	W/D	SE	5.11±0.05	5.13±0.07
Lagishetty, (2014)	Mice	0.50	3d	W/D†	SE	0.50±0.00	1.75±0.25‡
**Lung lavage protein**							
Holm (1987)	Rabbit	0.60	21d	BAL Protein (mg/kg)	SE	7.4±3.5	10.1±2
Van Klaveren, (1997)	Rat	0.60	7d	BAL albumin (ug/mL)	SD	215±85	328±99
Hesse (2004)	Mice	0.60	3d	BAL protein (µg/ml)†	SEM	100±50	100±25
**Lung pressure/volume measures**							
Hayatdavoudi,(1981)	Rat	0.60	7d	Airway pressure at TLC (cmH2O) ¶	SE	8.00±0.98	10.75±0.82‡
Hayatdavoudi,(1981)	Rat	0.60	7d	Air filled lung volume at 30 cmH2O (mL)	SE	15.30±.66	14.53±0.58
Hayatdavoudi,(1981)	Rat	0.60	7d	TLC (mL)	SE	14.43±1.99	12.60±2.04‡
Holm, (1987)	Rabbit	0.60	21d	TLC (mL) at 25 cmH2O	SE	88±4	96±6
Nickerson (1990)	Rabbit	0.60	21d	Lung volume (ml)	SE	68.7±2.8	66.2±5.4
Nelin, (2003)	Rat	0.50	2.4d	Pulmonary Compliance (ml/Torr)	SE	0.77±0.05	0.70±0.04
**Type-2 cell thymidine uptake**							
Hackney (1975)	Monkey	0.60	2d	Type-2 cell thymidine uptake index	NR	0.8	0.5
Hackney (1975)	Monkey	0.60	8d	Type-2 cell thymidine uptake index	NR	0.8	0.6
Van Klaveren, (1997)	Rat	0.60	7d	Type-2 cell thymidine uptake (counts/min)	SD	390±214	11,951±2,129§
**Other potential markers of Injury**							
Hayatdavoudi,(1981)	Rat	0.60	7d	Diffusing capacity (mL/min/Torr)	SE	0.21±0.04	0.18±0.04
Rister (1983)	Guinea Pigs	0.30	3.75d	Defects in MAC microtubules#	NR	20±3	24±3
Rister (1983)	Guinea Pigs	0.40	3.75d	Defects in MAC microtubules#	NR	20±3	21±3

Rister (1983)	Guinea Pigs	0.50	3.75d	Defects in MAC microtubules#	NR	20±3	17±3
Rister (1983)	Guinea Pigs	0.60	3.75d	Defects in MAC microtubules#	NR	20±3	34±4§
Holm, (1987)	Rabbit	0.60	21d	Type-2 cell PC uptake rate (nmoles/106 cells/h) †	SE	0.6±0.05	1.3±0.1‡
Holm, (1987)	Rabbit	0.60	21d	Type-2 cell DPPC uptake rate (nmoles/106 cells/h) †	SE	0.4±0.05	0.9±0.1‡
Holm, (1987)	Rabbit	0.60	21d	Type-2 cell diameter (um)	SE	9.6±0.05	13.0±0.9‡
Holm, (1987)	Rabbit	0.60	21d	Type-2 cell volume (um^3)	SE	467±76	1,214±21‡
Holm, (1987)	Rabbit	0.60	21d	Alveolar permeability (rate constant for solute flux)	SE	-7.0±2.0	-6.0±2.0
Van Klaveren, (1997)	Rat	0.60	7d	BAL volume (ml)	SD	30.0±3.5	29.6±4.2
Belik (2003)	Rat	0.60	14d	RV(g)/LV(g)	SE	0.26±0.00	0.29±0.01§
Belik (2003)	Rat	0.60	14d	PA force contraction to KCl (mN/mm^2)†	SE	18.0±2.0	9.9±0.1§
Belik (2003)	Rat	0.60	14d	PA force contraction to U466190 (mN/mm^2) #	SE	19.0±4.0	5.0±.1.0 §
Belik (2003)	Rat	0.60	14d	Airway force contraction to KCL (mN/mm^2)†	SE	18±2	9±0§
Belik (2003)	Rat	0.60	14d	Airway force contraction to U466190 (mN/mm^2)†#	SE	6±1	11±1§
Belik (2003)	Rat	0.60	14d	% PA relaxation to SNP from peak contraction†	SE	-90±0	-60±5§
Belik (2003)	Rat	0.60	14d	% PA relaxation to ACH from peak contraction†	SE	-45±5	0±5
Belik (2003)	Rat	0.60	14d	% Airway relaxation to SNP from peak contraction†	SE	-45±10	-40±2

*Measure descriptions and units are those provided in studies; † Means or medians and variances or interquartile ranges determined from figures; ‡ p≤0.05 as reported in studies; § p≤0.01 as reported in the studies; ¶-airway pressure in cmHg0 at total lung capacity in saline filled lungs; # % of alveolar macrophages with capped fluorescent distribution

ACH – acetylcholine; BAL – bronchoalveolar lavage; DPPC – dipamitoyl-phosphotidylcholine; g – grams; g/kg – gram/kilogram; KCL – potassium chloride; LM – light microscopy; LW – Lung weight; LW/BW – Lung weight/body weight; MAC – macrophage; mN – milli-Newtons; NA – not applicable; NR-Not Reported; PA – pulmonary artery; P/V – pressure/volume; PC – phosphatidyl choline; RV/LV – right ventricle weight to left ventricle weight ratio; SD – Standard Deviation; SE – Standard Error; SEM – Standard Error of the Mean; SNP – sodium nitroprusside; TLC – total lung capacity; U466190 – Thromboxane mimetic; W/D – Wet to dry ratio

Table S8. Results of immune response measures reported in O_2 only studies for groups exposed to FiO_2s≤0.60 and >0.21 or=0.21

Author (y)	Species	Increased FiO_2 Studied		Measures*		Reported Results for Groups	
		Level	Duration	Type	Variance	FiO_2=0.21	FiO_2 ≤0.60 and >0.21
**Antioxidant Activity**							
Hayatdavoudi, (1981)	Rat	0.60	7d	SOD (U/lung unit)	SE	3724±107	3881±40
Hayatdavoudi, (1981)	Rat	0.60	7d	G6PD (U/lung unit)	SE	3.4±0.2	3.7±0.2
Coursin, (1987)	Rat	0.50	7d	CAT (% of age matched FiO2=0.21 controls) †	SD	100±0	95±5
Coursin, (1987)	Rat	0.50	14d	CAT (% of age matched FiO2=0.21 controls) †	SD	100±0	125±20
Coursin, (1987)	Rat	0.50	21d	CAT (% of age matched FiO2=0.21 controls) †	SD	100±0	125±25
Coursin, (1987)	Rat	0.50	28d	CAT (% of age matched FiO2=0.21 controls) †	SD	100±0	150±25‡
Coursin, (1987)	Rat	0.50	42d	CAT (% of age matched FiO2=0.21 controls) †	SD	100±0	180±60s‡
Coursin, (1987)	Rat	0.50	7d	GPx (% of age matched FiO2=0.21 controls) †	SD	100±0	95±10
Coursin, (1987)	Rat	0.50	14d	GPx (% of age matched FiO2=0.21 controls) †	SD	100±0	115±15
Coursin, (1987)	Rat	0.50	21d	GPx (% of age matched FiO2=0.21 controls) †	SD	100±0	125±30
Coursin, (1987)	Rat	0.50	28d	GPx (% of age matched FiO2=0.21 controls) †	SD	100±0	110±10
Coursin, (1987)	Rat	0.50	42d	GPx (% of age matched FiO2=0.21 controls) †	SD	100±0	125±20
Coursin, (1987)	Rat	0.50	7d	NPSH (% of age matched FiO2=0.21 controls) †	SD	100±0	105±10
Coursin, (1987)	Rat	0.50	14d	NPSH (% of age matched FiO2=0.21 controls) †	SD	100±0	80±5‡
Coursin, (1987)	Rat	0.50	21d	NPSH (% of age matched FiO2=0.21 controls) †	SD	100±0	110±5
Coursin, (1987)	Rat	0.50	28d	NPSH (% of age matched FiO2=0.21 controls) †	SD	100±0	110±5
Coursin, (1987)	Rat	0.50	42d	NPSH (% of age matched FiO2=0.21 controls) †	SD	100±0	125±15‡
Van Klaveren, (1997)	Rat	0.60	7d	Lung GSH (nmol/mg)	SD	3.2±0.9	2.9±0.9
Van Klaveren, (1997)	Rat	0.60	7d	Lung SOD (U/mg lung)	SD	0.20±0.03	0.22±0.07
Van Klaveren, (1997)	Rat	0.60	7d	Lung GPx (mU/mg lung)	SD	0.05±0.02	0.24±0.03§
Gan (2011)	Rat	0.60	7d	DQH2 efflux rates during DQ infusion (µmol/min) †	SE	0.8±0.10	1.3±0.05‡
Gan (2011)	Rat	0.60	7d	DQH2 efflux rates during DQ + AA infusion (µmol/min) †	SE	1.2±0.05	1.51±0.01‡
Gan (2011)	Rat	0.60	7d	DQ efflux rates during DQH2 + DIC infusion (µmol/min) †	SE	0.95±0.05	1.2±0.10‡
Gan (2011)	Rat	0.60	7d	DQ efflux rates during DQH2 + DIC +ROT infusion (µmol/min) †	SE	1.00±0.05	1.4±0.10
Gan (2011)	Rat	0.60	7d	NQO1 activity (µmol min-1 lung-1) †	SE	26.48±1.67	34.75±1.98
Gan (2011)	Rat	0.60	7d	GSH + GSSG (µmol/lung) †	SE	2.34±0.17	3.09±0.14
Audi, (2012)	Rat	0.60	7d	HMPAQ retention without DEM (lung to background ratio) †	SE	4.0±0.05	5.9±0.1‡

Audi, (2012)	Rat	0.60	7d	HMPAQ retention with DEM (lung to background ratio) †	SE	2.9±0.1	4.5±0.1‡
Audi, (2012)	Rat	0.60	7d	GSH (µmol/g dry weight)	SE	10.15±0.56	12.07±0.46‡
Audi, (2012)	Rat	0.60	7d	GSH with DEM (µmol/g dry weight)	SE	0.39±0.22	1.89±0.26‡
Audi, (2012)	Rat	0.60	7d	GSSG (µmol/g dry weight)	SE	0.074±0.02	0.108±0.011
Audi, (2012)	Rat	0.60	7d	GSSG with DEM (µmol/g dry weight)	SE	ND	0.019±0.002
**BAL or Lung tissue MACs**							
Hayatdavoudi, (1981)	Rat	0.60	7d	BAL macrophages (total number of cells X 106)	SE	27±3	52±9‡
Hesse (2004)	Mice	0.60	3d	BAL macrophages (%)	SEM	98.8±0.5	97.7±1.0
**Changes in other Immune Response Parameters**							
Nelin (2003)	Rat	0.50	2.4d	2,3 DHBA (nmol/g lung) †	NR	0.25±0.01	0.24±0.01
Hesse (2004)	Mice	0.60	3d	BAL lymphocytes (%)	SEM	1.2±0.5	2.0±1.0
Hesse (2004)	Mice	0.60	3d	BAL neutrophils (%)	SEM	0.0±0.0	0.3±0.2
Hesse (2004)	Mice	0.60	3d	BAL TNFα (pg/mL) †	SEM	28±2	28±2
Hesse (2004)	Mice	0.60	3d	BAL Lung TBARS (nmol/mL) †	SEM	50±20	20±20
Lagishty (2014)	MIce	0.60	3d	Lung CLOCK/beta-actin mRNA (arbitrary units) †	SEM	6000±100	8000±100§
Lagishty (2014)	MIce	0.60	3d	Lung Bmal1/beta-actin mRNA (arbitrary units) †	SEM	6000±100	3000±100§
Lagishty (2014)	MIce	0.60	3d	Lung Cry1/beta-actin mRNA (arbitrary units) †	SEM	4000±100	3500±100
Lagishty (2014)	MIce	0.60	3d	Lung Cry2/beta-actin mRNA (arbitrary units) †	SEM	<100	1000±100§
Lagishty (2014)	MIce	0.60	3d	Lung Per1/beta-actin mRNA (arbitrary units) †	SEM	2500±100	5000±100§
Lagishty (2014)	MIce	0.60	3d	Lung Per2/beta-actin mRNA (arbitrary units) †	SEM	7000±100	6000±100

*Measure descriptions and units are those provided in studies; † Means or medians and variances or interquartile ranges determined from figures; ‡ p≤0.05 as reported in studies; § p≤0.01 as reported in studies

AA – Antimycin A; BAL – bronchioalveolar lavage; Bmal1 – aryl hydrocarbon receptor nuclear translocator-like protein; CAT – catalase activity; CLOCK – circadian locomotor output cycles kaput proteins; Cry – cryptochrome; DEM – diethyl maleate (depletes GSH); 2,3-DHBA – dihydroxyl benzoic acid (salicylate and OH product); DIC – dicumarol; GPx – Glutathione Peroxidase; DQH_2 – Durohydroquinone; DQ – duroquinone; GSH – glutathione; GSSG – oxidized glutathione; HMPAQ – Tc labeled hexamethylpropyleneamine oxide lung to background ratio (HMPAQ is trapped in the lung by GSH); MAC – macrophage; ND – Not detectable; NPSH – nonprotein sulfhydryl concentrations; NR – Not reported; Per – period homolog; ROT – rotenone; SA – salicylate; SOD – Superoxide Dismutase; TBARS – thiobarbituric acid reactive substrates; SE – Standard Error; SD – Standard deviation; TNF – tumor necrosis factor

Table S9. Results of lung injury and immune response measures reported in O2 only studies for groups exposed to FiO2s>0.60 or FiO2=0.21

Author (y)	Species	Fi02		Measures*		Reported Results for Groups	
		Level	Duration	Type	Variance	FiO2=0.21	FiO2>0.60
Lung Injury							
**Lung weights**							
Coursin, (1987)	Rat	0.65	4d	LW/BW (g/kg)†	SD	4.50±0.25	4.50±0.50
Coursin, (1987)	Rat	0.65	14d	LW/BW (g/kg)†	SD	4.40±0.25	4.05±0.10
Coursin, (1987)	Rat	0.65	21d	LW/BW (g/kg)†	SD	3.60±0.25	4.20±0.20
Coursin, (1987)	Rat	0.65	28d	LW/BW (g/kg)†	SD	3.60±0.50	3.80±0.20
Coursin, (1987)	Rat	0.65	42d	LW/BW (g/kg)†	SD	3.30±0.00	3.40±0.50
Coursin, (1987)	Rat	0.80	4d	LW/BW (g/kg)†	SD	4.50±0.25	4.50±0.40
Coursin, (1987)	Rat	0.80	14d	LW/BW (g/kg)†	SD	4.40±0.25	6.05±0.10§
Coursin, (1987)	Rat	0.80	21d	LW/BW (g/kg)†	SD	3.60±0.25	5.40±0.10§
Coursin, (1987)	Rat	0.80	28d	LW/BW (g/kg)†	SD	3.60±0.50	5.80±0.40‡
Coursin, (1987)	Rat	0.80	42d	LW/BW (g/kg)†	SD	3.30±0.00	5.10±0.10
Van Klaveren, (1997)	Rat	0.85	7d	W/D (gm H20/100 gm dry wgt)	SD	0.91±0.07	1.38±0.08§
Nelin, (2003)	Rat	0.90	2.4d	LW (g)	SE	1.23± 0.01	1.99±0.14‡
Audi, (2012)	Rat	0.95	2d	W/D	SE	5.11±0.05	5.24±0.08
Lagishetty, (2014)	Mice	0.75	3d	W/D†	SE	0.50±0.00	3.00±0.00§
Lagishetty, (2014)	Mice	1.0	1d	W/D†	SE	0.50±0.0	2.00±1.00‡
Lagishetty, (2014)	Mice	1.0	2d	W/D†	SE	0.50±0.0	4.50±1.00§
Lagishetty, (2014)	Mice	1.0	3d	W/D†	SE	0.50±0.0	7.00±1.00§
**BAL or lung lavage protein**							
Hesse (2004)	Mice	0.95	3d	BAL protein (µg/ml) †	SEM	100±50	1000±100§
Hayatdavoudi, (1981)	Rat	0.85	7d	BAL Total protein (mg)	SE	199±25	277±17
Van Klaveren, (1997)	Rat	0.85	7d	BAL protein (ml)	SE	30.0±3.5	21.6±2.7§
**Type-2 cell thymidine uptake**							
Hackney (1987)	Monkey	0.80	8d¶	Type 2 cells (labeled cells/1000 alveolar cells)	NR	0.7	15.3‡
Hackney (1987)	Monkey	0.80	4d¶	Type 2 cells (labeled cells/1000 alveolar cells)	NR	0.3	1.7‡
Van Klaveren, (1997)	Rat	0.85	7d	Type 2 cells (isolated cells x 106)	SE	5.9±1.7	9.8±0.5§
**Individual measures**							

Rister (1983)	Guinea Pigs	0.70	3.75d	Defects in MAC microtubules	NR	20±3	21±3
Rister (1983)	Guinea Pigs	0.80	3.75d	Defects in MAC microtubules	NR	20±3	17±3
Nelin, (2003)	Rat	0.90	2.4d	Pulmonary Compliance (ml/Torr)	SE	0.77±0.05	0.56±0.05‡
Immune Response							
**Anti-oxidants**							
Audi, (2012)	Rat	0.95	7d	HMPAQ retention without DEM (lung to background ratio) †	SE	4.0±0.05	13.5±0.5‡
Audi, (2012)	Rat	0.95	7d	HMPAQ retention with DEM (lung to background ratio) †	SE	2.9±0.1	6.0±0.1‡
Audi, (2012)	Rat	0.95	7d	GSH (µmol/g dry weight)	SE	10.15±0.56	14.17±0.46‡
Audi, (2012)	Rat	0.95	7d	GSH with DEM (µmol/g dry weight)	SE	0.39±0.22	1.34±0.26‡
Audi, (2012)	Rat	0.95	7d	GSSG (µmol/g dry weight)	SE	0.07±0.020	0.13±0.033
Audi, (2012)	Rat	0.95	7d	GSSG with DEM (µmol/g dry weight)	SE	ND	ND
Coursin, (1987)	Rat	0.65	7d	CAT (% of age matched controls) †	SD	100±0	105±10
Coursin, (1987)	Rat	0.65	14d	CAT (% of age matched controls) †	SD	100±0	125±15
Coursin, (1987)	Rat	0.65	21d	CAT (% of age matched controls) †	SD	100±0	175±25
Coursin, (1987)	Rat	0.65	28d	CAT (% of age matched controls) †	SD	100±0	190±30
Coursin, (1987)	Rat	0.65	42d	CAT (% of age matched controls) †	SD	100±0	250±50‡
Coursin, (1987)	Rat	0.80	4d	CAT (% of age matched controls) †	SD	100±0	199±10§
Coursin, (1987)	Rat	0.80	7d	CAT (% of age matched controls) †	SD	100±0	225±35‡
Coursin, (1987)	Rat	0.80	14d	CAT (% of age matched controls) †	SD	100±0	275±25§
Coursin, (1987)	Rat	0.80	21d	CAT (% of age matched controls) †	SD	100±0	190±15‡
Coursin, (1987)	Rat	0.80	28d	CAT (% of age matched controls) †	SD	100±0	225±25§
Coursin, (1987)	Rat	0.80	42d	CAT (% of age matched controls) †	SD	100±0	275±100
Coursin, (1987)	Rat	1.0	4d	CAT (% of age matched controls) †	SD	100±0	233±33§
Coursin, (1987)	Rat	0.65	7d	GPx (% of age matched controls) †	SD	100±0	125±20
Coursin, (1987)	Rat	0.65	14d	GPx (% of age matched controls) †	SD	100±0	105±15
Coursin, (1987)	Rat	0.65	21d	GPx (% of age matched controls) †	SD	100±0	175±15§
Coursin, (1987)	Rat	0.65	28d	GPx (% of age matched controls) †	SD	100±0	125±10‡
Coursin, (1987)	Rat	0.65	42d	GPx (% of age matched controls) †	SD	100±0	145±5‡
Coursin, (1987)	Rat	0.80	4d	GPx (% of age matched controls) †	SD	100±0	135±10‡
Coursin, (1987)	Rat	0.80	7d	GPx (% of age matched controls) †	SD	100±0	150±15§
Coursin, (1987)	Rat	0.80	14d	GPx (% of age matched controls) †	SD	100±0	130±10
Coursin, (1987)	Rat	0.80	21d	GPx (% of age matched controls) †	SD	100±0	140±15‡
Coursin, (1987)	Rat	0.80	28d	GPx (% of age matched controls) †	SD	100±0	145±5§
Coursin, (1987)	Rat	0.80	42d	GPx (% of age matched controls) †	SD	100±0	140±20
Coursin, (1987)	Rat	1.0	4d	GPx (% of age matched controls) †	SD	100±0	160±15§

Coursin, (1987)	Rat	0.65	7d	NPSH (% of age matched controls) †	SD	100±0	125±5§
Coursin, (1987)	Rat	0.65	14d	NPSH (% of age matched controls) †	SD	100±0	80±5‡
Coursin, (1987)	Rat	0.65	21d	NPSH (% of age matched controls) †	SD	100±0	145±5§
Coursin, (1987)	Rat	0.65	28d	NPSH (% of age matched controls) †	SD	100±0	125±5‡
Coursin, (1987)	Rat	0.65	42d	NPSH (% of age matched controls) †	SD	100±0	145±10§
Coursin, (1987)	Rat	0.80	4d	NPSH (% of age matched controls) †	SD	100±0	100±5
Coursin, (1987)	Rat	0.80	7d	NPSH (% of age matched controls) †	SD	100±0	155±25§
Coursin, (1987)	Rat	0.80	14d	NPSH (% of age matched controls) †	SD	100±0	125±5‡
Coursin, (1987)	Rat	0.80	21d	NPSH (% of age matched controls) †	SD	100±0	120±5
Coursin, (1987)	Rat	0.80	28d	NPSH (% of age matched controls) †	SD	100±0	145±5§
Coursin, (1987)	Rat	0.80	42d	NPSH (% of age matched controls) †	SD	100±0	160±15
Coursin, (1987)	Rat	1.0	4d	NPSH (% of age matched controls) †	SD	100±0	125±10§
Coursin, (1987)	Rat	0.80	4d	SOD (% of age matched controls)	SD	100±0	111±17
Coursin, (1987)	Rat	0.80	7d	SOD (% of age matched controls)	SD	100±0	148±19‡
Coursin, (1987)	Rat	0.80	14d	SOD (% of age matched controls)	SD	100±0	114±13
Coursin, (1987)	Rat	0.80	21d	SOD (% of age matched controls)	SD	100±0	127±10‡
Coursin, (1987)	Rat	0.80	28d	SOD (% of age matched controls)	SD	100±0	98±6
Coursin, (1987)	Rat	0.80	42d	SOD (% of age matched controls)	SD	100±0	111±7
Coursin, (1987)	Rat	1.0	4d	SOD (% of age matched controls)	SD	100±0	100±23
Coursin, (1987)	Rat	0.80	4d	GSSG (% of age matched controls)	SD	100±0	121±21
Coursin, (1987)	Rat	0.80	7d	GSSG (% of age matched controls)	SD	100±0	121±15
Coursin, (1987)	Rat	0.80	14d	GSSG (% of age matched controls)	SD	100±0	82±5§
Coursin, (1987)	Rat	0.80	21d	GSSG (% of age matched controls)	SD	100±0	95±15
Coursin, (1987)	Rat	0.80	28d	GSSG (% of age matched controls)	SD	100±0	99±6
Coursin, (1987)	Rat	0.80	42d	GSSG (% of age matched controls)	SD	100±0	98±6
Coursin, (1987)	Rat	1.0	4d	GSSG (% of age matched controls)	SD	100±0	135±22‡
Hayatdavoudi, (1981)	Rat	0.60	7d	SOD (U/lung unit)	SE	4387±189	7088±302§
Hayatdavoudi, (1981)	Rat	0.60	7d	G6PD (U/lung unit)	SE	3.1±0.4	8.5±1.1§
Van Klaveren, (1997)	Rat	0.85	7d	Tissue GSH	SD	0.85±0.05	0.80±0.15
Van Klaveren, (1997)	Rat	0.85	7d	Lung GSH	SD	3.2±0.9	4.6±1.1
Van Klaveren, (1997)	Rat	0.85	7d	Lung SOD	SD	0.20±0.03	0.91±0.07
Van Klaveren, (1997)	Rat	0.85	7d	Lung GPx	SD	0.05±0.02	0.98±0.22
**BAL or lung tissue MACs**							
Hesse (2004)	Mice	0.95	3d	BAL macrophages (%)	SEM	98.8±0.5	86.2±1.8‡
Hackney (1987)	Monkey	0.80	8d¶	BAL Macrophages (labeled cells/1000 alveolar cells)	Mean	0.6	2.8

Hackney (1987)	Monkey	0.80	4d¶	BAL Macrophages (labeled cells/1000 alveolar cells)	Mean	0.5	0.5
**BAL PMNs**							
Hesse (2004)	Mice	0.95	3d	BAL neutrophils (%)	SEM	0.0±0.0	4.6±1.0‡
**BAL or lung lymphocytes**							
Hesse (2004)	Mice	0.60	3d	BAL lymphocytes (%)	SEM	1.2±0.5	9.2±0.9‡

*Measure descriptions and units are those provided in studies; † Means or medians and variances or interquartile ranges determined from figures; ‡ p≤0.05 as reported in studies; § p≤0.01 as reported in studies

BAL – bronchioalveolar lavage; CAT – catalase activity; DEM – GSH depleter diethyl maleate; GSH – glutathione; GPx – Glutathione Peroxidase; GSSG – oxidized glutathione; HMPAQ – Hexamethylpropyleneamine oxime lung to background ratio; LV – Lung Volume; LW – Lung weight; MAC – macrophage; ND – Not detectable; NPSH – nonprotein sulfhydryl concentrations; NR – Not Reported; PA – pulmonary artery; PMN – polymorphonuclear leukocytes; P/V – pressure/volume; SE – Standard Error; SEM – Standard Error of the Mean; TLC – total lung capacity; SD – Standard Deviation; SOD – Superoxide Dismutase; G6PD – glucose-6-phosphate dehydrogenase; W/D – Wet to dry ratio

Table S10. Quality of evidence, adapted from SYRCLE

Author/year	Sample size calculation	Randomization procedure	Groups similar at baseline (weight and/or age)	Blinding of challenges	Blinding to results assessment	Animals removed from study	Random animal housing
O_2+nonO_2 Challenge Studies							
Cheney (1980)	UC	UC	YES	NO	UC	YES	UC
Rinaldo (1985)	UC	UC	YES	NO	UC	UC	UC
Garner (1988)	UC	UC	YES	NO	UC	UC	UC
Cantor (1990)	UC	UC	YES	NO	UC	UC	UC
Knight (2000)	UC	UC	YES	NO	UC	YES	UC
Nara (2004)	UC	UC	UC	NO	UC	UC	UC
Sun (2006)	UC	YES	YES	NO	UC	UC	UC
Aggarwal (2010)	UC	UC	YES	NO	UC	UC	UC
Rodriguez-Gonzalez (2014)	YES	YES	YES	NO	UC	UC	UC
Garcia-Laorden (2020)	UC	YES	YES	NO	UC	UC	UC
O_2 only Studies							
Hackney (1975)	UC	UC	UC	NO	UC	YES	UC
Hayatdavoudi (1981)	UC	UC	YES	NO	UC	UC	UC
Rister (1983)	UC	UC	UC	NO	UC	UC	UC
Coursin (1987)	UC	UC	YES	NO	UC	UC	UC
Holm (1987)	UC	UC	YES	NO	UC	UC	UC
Nickerson (1990)	UC	YES	YES	NO	UC	UC	UC
Nylen (1993)	UC	UC	YES	NO	UC	NO	UC
Van Klaveren (1997)	UC	UC	YES	NO	UC	YES	UC
Belik (2003)	YES	UC	YES	NO	UC	UC	UC
Nelin (2003)	UC	UC	YES	NO	UC	UC	UC
Hesse (2004)	UC	YES	YES	NO	UC	YES	UC
Gan (2011)	UC	UC	YES	NO	UC	UC	UC
Audi (2012)	UC	UC	YES	NO	UC	UC	UC
Lagishetty (2014)	UC	UC	YES	NO	UC	YES	UC

SYRCLE – Systematic Review Center for Laboratory Experimentation; UC – unclear
