# Supplementary material for: Effect of low-to-moderate hyperoxia on lung injury in preclinical animal models: a systematic review and meta-analysis
Source: Intensive Care Med Exp. 2023 Apr 24;11:22. doi: 10.1186/s40635-023-00501-x (PMC10122981; doi:10.1186/s40635-023-00501-x)
Supplement: Supplementary file 3 — Additional file 3. Search strategy used for PUBMED, Web of Science, and EMBASE. [file 40635_2023_501_MOESM3_ESM.docx]

9/27/2021

**FINAL SEARCH STRING PUBMED = 9,844 citations**

**((sepsis[majr] OR sepsis[tiab] OR septic[tiab] OR septicemia[tiab] OR bacterial infections[majr] OR bacterial infection*[tiab] OR critical illness[majr] OR critical illness*[tiab] OR critically ill[tiab] OR critical care[majr] OR critical care[tiab] OR endotoxins[majr] OR endotoxin*[tiab] OR endotoxemia[majr] OR endotoxemia[tiab] OR lipopolysaccharides[majr] OR lipopolysaccharide*[tiab] OR systemic inflammation*[tiab] OR Bacterial Infections and Mycoses[Mesh] OR Virus Diseases[Mesh] OR virus disease*[tiab] OR viral infection*[tiab] OR viral illness*[tiab] OR virus[tiab] OR virus infection*[tiab] OR virus illness*[tiab] OR lung challenges[tiab] OR lung injury models[tiab] OR acute lung injury[tiab] OR Lung diseases, fungal[Mesh] OR pneumonia, viral[Mesh] OR viral pneumonia[tiab] OR pneumonia, bacterial[Mesh] OR bacterial pneumonia[tiab] OR lung[majr]) AND (oxygen support[tiab] OR oxygen therapy[tiab] OR oxygen administration[tiab] OR Oxygen exposure[tiab] OR oxygen treatment[tiab] OR hyperoxia[majr] OR hyperoxia[tiab] OR respiratory support[tiab] OR ventilation therapy[tiab] OR ventilation support[tiab] OR oxygen inhalation therapy[tiab] OR respiration, artificial[Mesh] OR artificial respiration[tiab] OR hypoxia[Mesh] OR hypoxia[tiab])) AND (Animals[Mesh:NoExp] OR mouse[tiab] OR mice[tiab] OR rats[tiab] OR dogs[tiab] OR canine[tiab] OR guinea pigs[tiab] OR swine[tiab] OR pigs[tiab] OR primate*[tiab] OR rabbit*[tiab] OR monkey*[tiab] OR preclinical[tiab] OR non-human primate[tiab] OR mammal*[tiab])**

**FINAL SEARCH STRING WEB OF SCIENCE = 2,936 citations**

TOPIC: (sepsis OR septic OR septicemia OR “bacterial infection*” OR “critical illness*” OR “critically ill” OR “critical care” OR endotoxin* OR endotoxemia OR lipopolysaccharide* OR systemic inflammation OR bacterial infection* OR mycoses OR “virus disease*” OR “viral infection*” OR “viral illness*” OR virus OR “virus infection*” OR “acute respiratory distress” OR “respiratory distress syndrome” OR “respiratory tract infections” OR “fungal lung disease*” OR pneumonia)

AND

TOPIC: (Animals OR mouse OR mice OR rat* OR dog* OR canine OR “guinea pig*” OR swine OR pig* OR primate* OR rabbit* OR monkey* OR preclinical OR “non-human primate” OR mammal*)

AND

TOPIC: (“oxygen support” OR “oxygen therapy” OR “oxygen administration” OR “oxygen exposure” OR “oxygen treatment” OR hyperoxia OR “respiratory support” OR “ventilation therapy” OR “ventilation support” OR “oxygen inhalation therapy” OR “oxygen concentration” OR “high oxygen” OR “oxygen tolerance” OR “oxygen level”)

**Final Search String EMBASE = 1,589**

(**'oxygen support'** OR **'oxygen therapy'**/exp OR **'oxygen therapy'** OR **'oxygen administration'**/exp OR **'oxygen administration'** OR **'oxygen exposure'**/exp OR **'oxygen exposure'** OR **'oxygen treatment'**/exp OR **'oxygen treatment'** OR **'hyperoxia'**/exp OR **hyperoxia** OR **'respiratory support'**/exp OR **'respiratory support'** OR **'ventilation therapy'** OR **'ventilation support'** OR **'oxygen inhalation therapy'**/exp OR **'oxygen inhalation therapy'** OR **'oxygen concentration'**/exp OR **'oxygen concentration'** OR **'high oxygen'** OR **'oxygen tolerance'** OR **'oxygen level'**) AND (**'sepsis'**/exp OR **sepsis** OR **septic** OR **'septicemia'**/exp OR **septicemia** OR **'bacterial infection'**/exp OR **'bacterial infection'** OR **'critical illness'**/exp OR **'critical illness'** OR **'critical illnesses'** OR **'critically ill'**/exp OR **'critically ill'** OR **'critical care'**/exp OR **'critical care'** OR **'endotoxins'**/exp OR **endotoxins** OR **'endotoxin'**/exp OR **endotoxin** OR **'endotoxemia'**/exp OR **endotoxemia** OR **'lipopolysaccharides'**/exp OR **lipopolysaccharides** OR **'lipopolysaccharide'**/exp OR **lipopolysaccharide** OR **'systemic inflammation'**/exp OR **'systemic inflammation'** OR **'bacterial infections'**/exp OR **'bacterial infections'** OR **'mycoses'**/exp OR **mycoses** OR **'virus diseases'**/exp OR **'virus diseases'** OR **'virus disease'**/exp OR **'virus disease'** OR **'viral infection'**/exp OR **'viral infection'** OR **'viral illness'** OR **'virus'**/exp OR **virus** OR **'virus infections'** OR **'virus illnesses'** OR **'acute respiratory distress'**/exp OR **'acute respiratory distress'** OR **'respiratory distress syndrome'**/exp OR **'respiratory distress syndrome'** OR **'respiratory tract infections'**/exp OR **'respiratory tract infections'** OR **'fungal lung disease'** OR **'fungal lung diseases'**/exp OR **'fungal lung diseases'** OR **'pneumonia'**/exp OR **pneumonia**) AND (**'animals'**/exp OR **animals** OR **'mouse'**/exp OR **mouse** OR **'mice'**/exp OR **mice** OR **'rat'**/exp OR **rat** OR **'dog'**/exp OR **dog** OR **'dogs'**/exp OR **dogs** OR **'canine'**/exp OR **canine** OR **'guinea pig'**/exp OR **'guinea pig'** OR **'guinea pigs'**/exp OR **'guinea pigs'** OR **'swine'**/exp OR **swine** OR **'pig'**/exp OR **pig** OR **'pigs'**/exp OR **pigs** OR **'primate'**/exp OR **primate** OR **'primates'**/exp OR **primates** OR **'rabbit'**/exp OR **rabbit** OR **'rabbits'**/exp OR **rabbits** OR **'monkeys'**/exp OR **monkeys** OR **'monkey'**/exp OR **monkey** OR **preclinical** OR **'non-human primate'** OR **'mammal'**/exp OR **mammal** OR **'mammals'**/exp OR **mammals**) Limits: Embase; Not Medline
