# Supplementary material for: Effect of low-to-moderate hyperoxia on lung injury in preclinical animal models: a systematic review and meta-analysis
Source: Intensive Care Med Exp. 2023 Apr 24;11:22. doi: 10.1186/s40635-023-00501-x (PMC10122981; doi:10.1186/s40635-023-00501-x)
Supplement: Supplementary file 4 — Additional file 4. Extraction form utilitzed by authors for O2 only studies and O2 with non-O2 studies. [file 40635_2023_501_MOESM4_ESM.docx]

Author:

Year of Publication:

Country:

Other studies in this review (Y/N and list):

Potentially important reference studies:

Oxygen only versus Oxygen with another challenge (What type):

General question/s being asked:

Animals

Species:

Subspecies/strain (if applicable):

Age:

Sex:

Weight:

Goals/outcomes assessed of Individual Experiments:

Experiment # 1

Experiment # 2

Experiment # 3

Experiment # 4

O2 Only Challenge Study

Level, duration, and ATM of FiO2 administered >21% ≤60% (repeat for each group and experiment):

Level and duration of control exposure (repeat for each experiment):

Mechanical ventilation (Y/N):

Numbers of animals studied

Oxygen Exposed Control

FiO2/duration #1 FiO2/duration #2

Experiment # 1

Experiment # 2

Experiment # 3

Experiment # 4

Total animal

numbers

O2 Only Challenge Study

Outcomes/Results [Describe as animal numbers (number with/total) or mean/median with variances for each group):

Oxygen Exposed Groups Control

List outcomes/results

Under each experiment

Experiment # 1

Experiment # 2

Experiment # 3

Experiment # 4

Others

O2 with Non-O2 Challenge Study

Non-O2 Challenge description:

Level, duration, and ATM of FiO2 administered >21% ≤60% and type, dose and timing of non-O2 challenge (repeat for each group and experiment):

Level and duration of control exposure and dose and timing of non-O2 challenge (repeat for each experiment):

Mechanical ventilation (Y/N):

Antibiotics vs. other treatment (fluids etc.) (Y/N):

Numbers of animals studied - describe conditions and numbers for each experiment:

Oxygen Exposed Non-O2 exposed

With or without non-O2 challenge With or without non-O2 challenge

Experiment # 1

Experiment # 2

Experiment # 3

Experiment # 4

Total animal

numbers

O2 with Non-O2 Challenge Study

Outcomes/Results [Describe as animal numbers (number with/total) or mean/median with variances for each group):

Oxygen Exposed Non-O2 exposed

With or without non-O2 challenge With or without non-O2 challenge List outcomes/results

Under each experiment

Experiment # 1

Experiment # 2

Experiment # 3

Experiment # 4

Others

Analysis

Statistical tests used:

Study Quality/Risk of Bias – (See Busch paper which used an adapted “Systematic Review Centre for Laboratory Animal Experimentation (SYRCLE) grading system) (yes/no/other)

Primary outcome clearly listed:

Sample size calculations/Power analysis:

Randomization of challenges and treatments:

Confirmation of baseline similarity of study group animals (e.g. age, weight):

Blinding of challenges, treatments, and outcome assessments:

Randomized between groups:
